# Unveiling some FDA-approved drugs as inhibitors of the store-operated Ca^2+^ entry pathway

**DOI:** 10.1038/s41598-017-13343-x

**Published:** 2017-10-16

**Authors:** Saifur Rahman, Taufiq Rahman

**Affiliations:** Department of Pharmacology, University of Cambridge, Tennis Court Road Cambridge, UK

## Abstract

The store-operated calcium entry (SOCE) pathway is an important route for generating cytosolic Ca^2+^ signals that regulate a diverse array of biological processes. Abnormal SOCE seem to underlie several diseases that notably include allergy, inflammation and cancer. Therefore, any modulator of this pathway is likely to have significant impact in cell biology under both normal and abnormal conditions. In this study, we screened the FDA-approved drug library for agents that share significant similarity in 3D shape and surface electrostatics with few, hitherto best known inhibitors of SOCE. This has led to the identification of five drugs that showed dose-dependent inhibition of SOCE in cell-based assay, probably through interacting with the Orai1 protein which effectively mediates SOCE. Of these drugs, leflunomide and teriflunomide could suppress SOCE significantly at clinically-relevant doses and this provides for an additional mechanism towards the therapeutic utility of these drugs as immunosuppressants. The other three drugs namely lansoprazole, tolvaptan and roflumilast, were less potent in suppressing SOCE but were more selective and thus they may serve as novel scaffolds for future development of new, more efficacious SOCE inhibitors.

## Introduction

Ca^2+^ is a universal and versatile intracellular messenger which regulates almost every aspect of cellular life ranging from fertilisation to cell death. Generation of Ca^2+^ signals involves a transient increase in the cytosolic free Ca^2+^ concentration from the resting low (≤500 nM) level up to ~1 μM^1^. To do this, cells rely on stimulus-dependent release of Ca^2+^ from the internal stores (e.g. the endoplasmic reticulum, ER) as well as on Ca^2+^ entry from the extracellular space through various plasma membrane (PM)-localised, Ca^2+^ permeable ion channels that can be either ligand- or voltage-gated. However, for many cells a major source of cytosolic Ca^2+^ signals happens to be a distinct and unique Ca^2+^ entry mechanism namely the store-operated Ca^2+^ entry (SOCE). SOCE occurs when intracellular Ca^2+^ stores are rapidly depleted, which then triggers a more sustained Ca^2+^ entry via PM-localised Orai1 channels (also known as Ca^2+^ release-activated Ca^2+^ channels, CRAC channels). The latter are activated upon binding with the stromal interaction molecule-1 (STIM1) proteins which are expressed within the ER membrane and effectively ‘sense’ the Ca^2+^ drop within the ER lumen. Although SOCE was first identified in mast cells and mostly studied in this and other non-excitable cells, evidence amass that such Ca^2+^ entry pathway probably exists in all kind of cells including even the excitable ones^2^.

SOCE is traditionally well known to serve as the major route for replenishing the depleted intracellular Ca^2+^ stores. Apart from such important housekeeping task, evidence continues to emerge that SOCE can also deliver spatio-temporally complex Ca^2+^ signals for regulating some more specific biological processes such as exocytosis, mitochondrial metabolism, gene expression, cell growth and proliferation^3^. In recent years, aberrant Orai1 channel activity has been noted in several human diseases, including severe combined immunodeficiency disorders, allergy, thrombosis, acute pancreatitis, inflammatory bowel disease, rheumatoid arthritis and cancer^4^. Thus there have been legitimate active interests in the academia and industries for developing specific inhibitors of SOCE/CRAC channels. Although a number of small molecules have emerged as SOCE inhibitors by now^3–6^, most of them by far have not reached clinical trials, primarily owing to their inadequate selectivity and high toxicity. It is however encouraging to note that a member of the CalciMedica (CM4620) series has recently reached Phase I clinical trials with intended use for treating acute pancreatitis^7^. Nevertheless, the need for identifying new scaffolds against SOCE/CRAC channels remains still valid for future development of more specific inhibitors with improved potency, greater selectivity and known mechanism of action.

Given the crucial involvement of the SOCE-derived Ca^2+^ signals in the regulation of some specific cellular processes mentioned above, any modulator of this pathway is likely to have substantial effects on cell biology under normal as well as pathological conditions. Thus, there can be drug molecules possessing hitherto undisclosed capacity for modulating SOCE at therapeutically relevant doses. Such property could potentially contribute to their clinical benefits within a polypharmacological context or could perhaps explain some of their side effects. In the present study, we aimed at identifying any such drug(s) using a ligand-based *in silico* approach. For this, we have exploited the structures of few best known SOCE inhibitors as baits and virtually screened FDA-approved drug library to find drugs that share significant similarities in 3D shape and electrostatics with these baits and thus are likely to phenocopy them. Indeed through subsequent bioassay of the shortlisted drug hits, we identified five drugs that dose-dependently suppress SOCE and these drugs do not have any previous report of such action. For two of the five drugs, significant inhibition of SOCE seems to occur at a clinically relevant dose and thus this could contribute to their therapeutic utility for indicated conditions. The other three drugs can serve as novel scaffold for future development of novel series of SOCE inhibitors. Our finding thus re-validates the utility of such computational approach in drug repurposing endeavour as indicated before^8,9^.

## Results

### Ligand-based virtual screening and identification of hits for bioassay

We first sought out to evaluate the performance of our *in silico* protocol and for this, we screened conformer libraries generated from the TOCRIS™ (http://zinc15.docking.org/catalogs/tocris/) and the MedchemExpress™ (http://zinc15.docking.org/catalogs/mce/) libraries using BTP2 and Pyr6 as baits, respectively. We chose these libraries as BTP2 and Pyr6 are enlisted as purchasable items in TOCRIS and the MedChemExpress databases, respectively. It was reassuring to note that BTP2 and Pyr6 ranked the top in both ROCS based primary screening as well as in EON based re-ranking of ROCS outputs from screening of TOCRIS and MedChemExpress libraries, respectively (Supplementary Tables 1 and 2). We therefore went ahead with our protocol to screen a conformer library generated from the eDrugs3D database^10^ that contains all FDA-approved drugs. As baits, we used Synta66 and AnCoA4, in addition to BTP2 and Pyr6 (Supplementary Fig. 1). After visual inspection of each hit for electrostatic similarity and considering their ranks in ROCS and EON based outputs, availability and any previous record of having any effect on cellular calcium signals, we purchased total 11 drugs (Supplementary Table 3). Of these, we could not screen flutamide as it was coloured and incompatible with our calcium indicator. So the final list of 10 drugs that were tested in single cell Ca^2+^ imaging include leflunomide, teriflunomide, tolvaptan, conivaptan, omeprazole, lansoprazole, rufinamide, prazosin, terazosin and roflumilast.

### Evaluation of the effects of chosen drugs on the store-operated calcium entry (SOCE)

**Figure 1:**
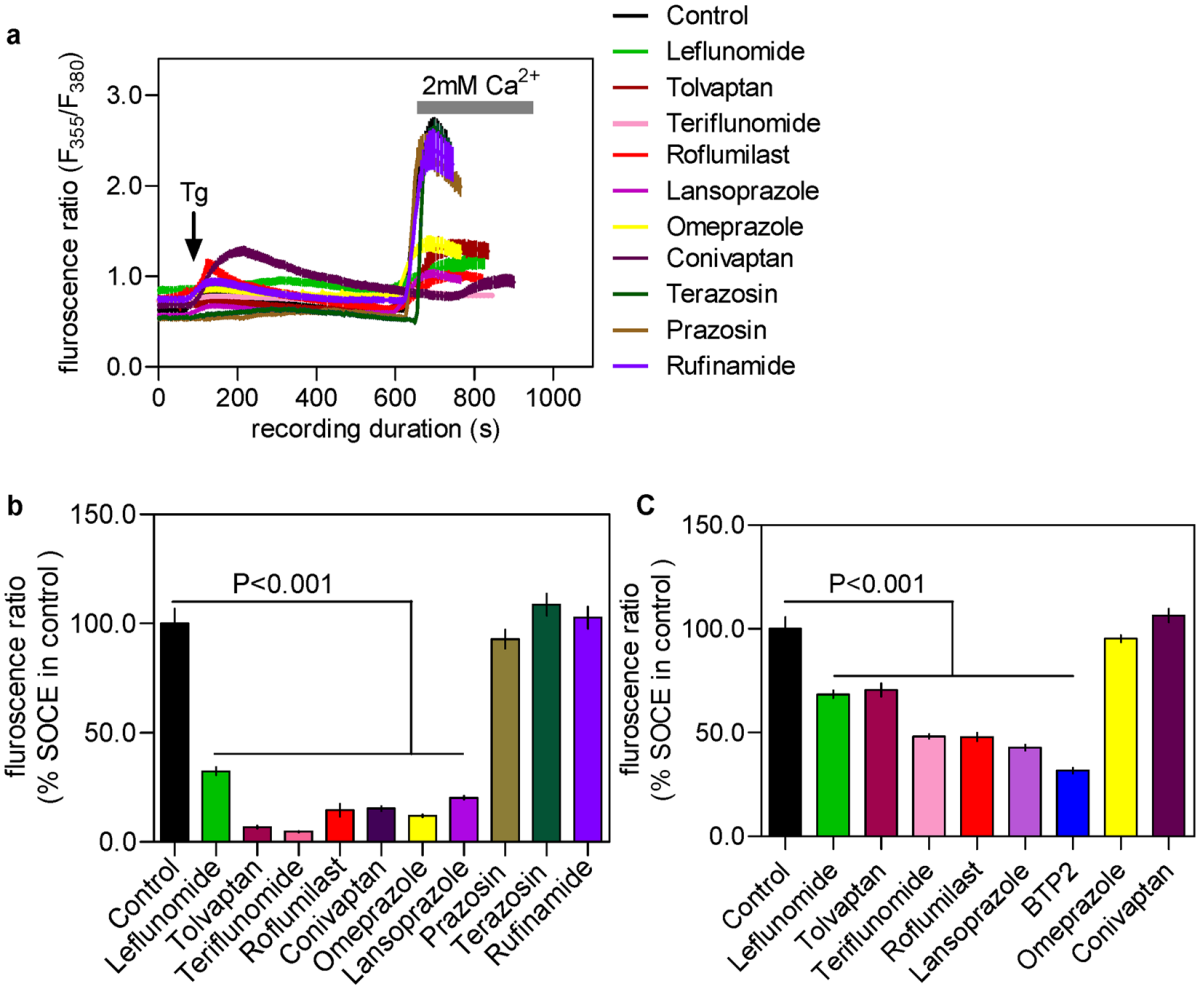
Evaluation of the effects of chosen drugs on the store-operated calcium entry (SOCE) in RBL-1 cells triggered by thapsigargin using Fura-2 based Ca^2+^ imaging experiments. (**a**) Sample traces representing Ca^2+^ signals (indicated by Fura-2 fluorescence ratio) triggered by adding 2 μM of thapsigargin (Tg) to RBL-1 cells with or without pre-treatment of the chosen drugs (shown in various coloured lines, each at 100 μM dose). (**b**) Histograms showing the peak SOCE levels triggered by Tg in control cells and cells treated with 100 μM of each drug. (**c**) Histograms showing the peak SOCE levels triggered by Tg in control cells and cells pre-treated with each drug at 10 μM dose. Each value (mean ± SEM) was derived from 3–5 individual experiments and a total of 30–80 cells. The statistical comparison among the values for all conditions was determined using one way ANOVA followed by Dunnett’s test.

To evaluate the effect of the purchased drugs on SOCE, we used Fura-2 based Ca^2+^ imaging^11,12^ primarily with RBL-1 cells which are widely-used for studying SOCE. These cells were first treated, in absence of extracellular Ca^2+^, with thapsigargin (Tg) that passively depletes the intracellular Ca^2+^ store by inhibiting the serco-endoplasmic Ca^2+^-ATPase (SERCA). SOCE was evident in these cells soon after the addition of 2 mM Ca^2+^ to the extracellular (bath) solution. We first screened the drugs at 100 μM and at this concentration most of the purchased drugs showed substantial (by ≥60%) suppression of SOCE. Only prazosin, terazosin and rufinamide had no effect on SOCE at this high concentration and therefore discarded for further investigation (Fig. 1a,b).

**Figure 2:**
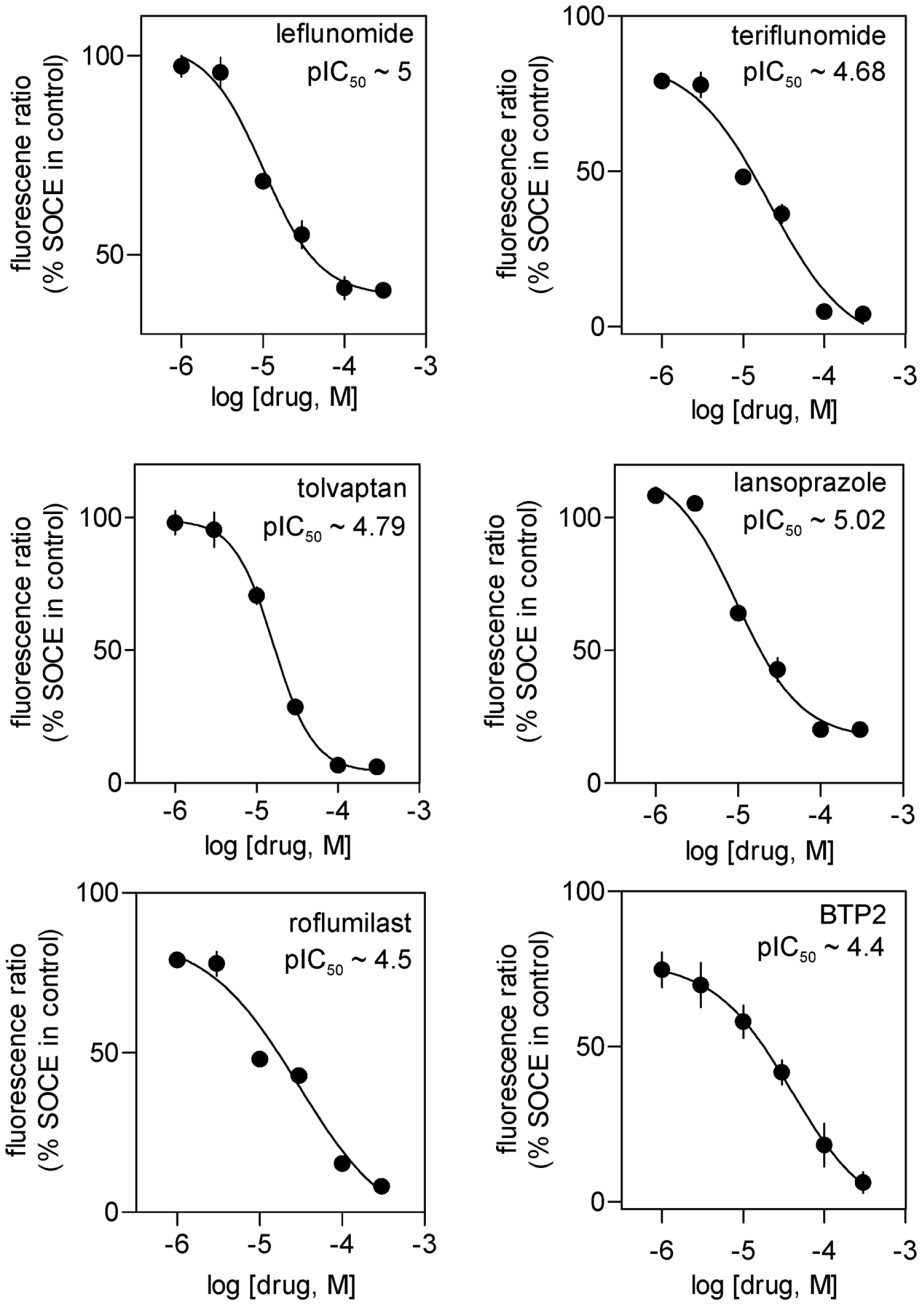
Concentration-response curves demonstrating the dose-dependence of inhibitory effect of indicated drugs and BTP2 on the SOCE in RBL-1 cells triggered by Thapsigargin. SOCE was denoted by the peak Fura-2 fluorescence ratio following Ca^2+^ add back during Ca^2+^ imaging experiment of RBL-1 cells loaded with Fura-2AM. Each value (mean ± SEM) was derived from 3–5 individual experiments and a total of 60–100 cells.

In order to identify the more potent drugs from the list, we then screened all the remaining drugs at 10-fold lower concentration (10 μM) and apart from omeprazole and conivaptan, all drugs retained their ability to inhibit SOCE significantly at this concentration (Fig. 1c). For those 5 drugs which significantly suppressed SOCE at 10 μM, we carried out complete concentration-response studies with a view to evaluating the concentration-dependence of their action as well as determining the half-maximal inhibitory concentration (IC_50_) for each drug. As can be seen in Fig. 2, all 5 drugs as well as the positive control, BTP2 showed dose-dependent suppression of SOCE over the concentration range of 300 μM to 10 μM. At concentration below 10 μM, none of the drugs under the standard experimental protocol (~20 min pre-incubation before the Ca^2+^ add back) manifested any significant SOCE-inhibitory property. Since the maximum concentration of the drugs used in the study (i.e. 300 μM) was fairly high, we also assessed the possible toxic effects of these drugs at such high concentration using a widely-used, propidium iodide-based cell viability assay^13^. It was reassuring that none of drugs even at their highest concentration used in our calcium imaging, was toxic to RBL-1H cells (Supplementary Fig. 2).

**Figure 3:**
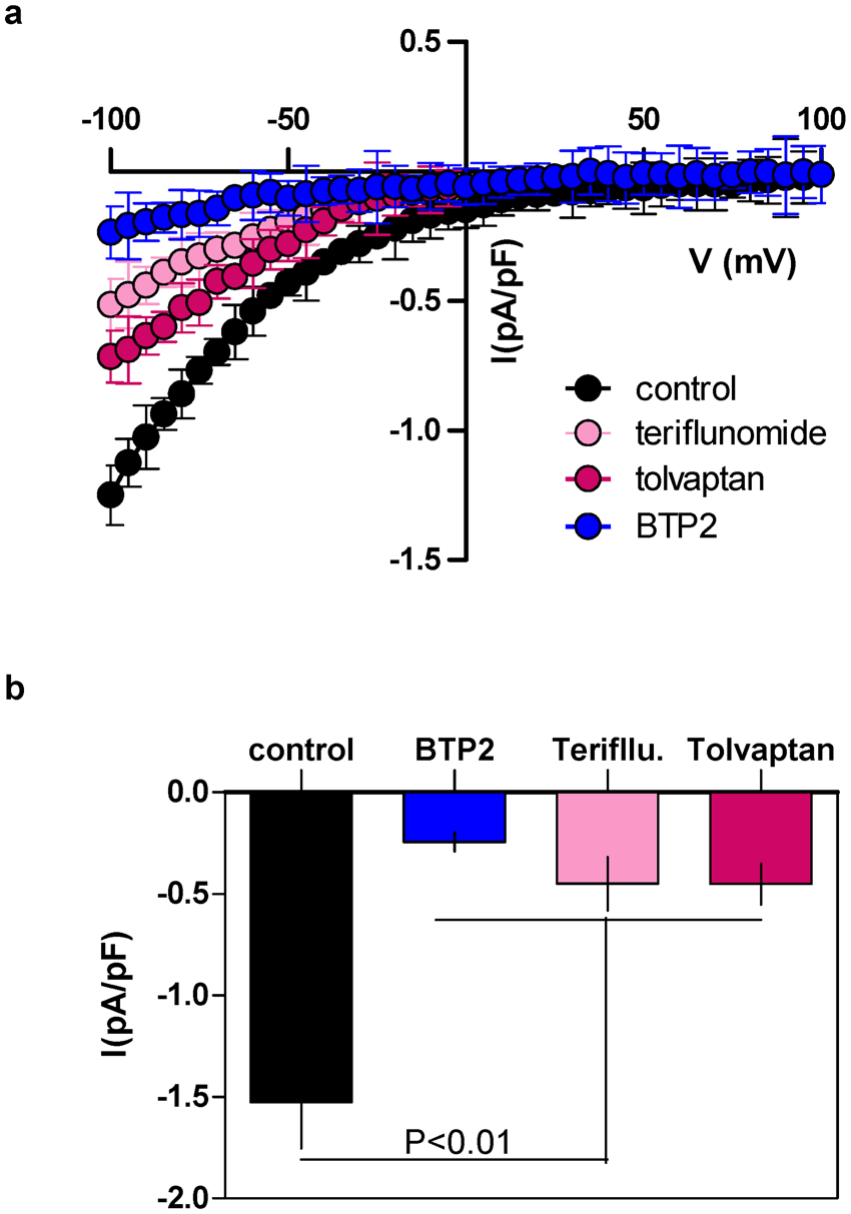
Effects of selected compounds on CRAC current. (**a**) RBL-1 cells were pre-treated for ~20 min with BTP2 (10 μM), teriflunomide (20 μM) and tolvaptan (20 μM) whilst the control cells received DMSO. Representative current-voltage plots (mean ± S.E.M) from whole-cell recording of at least four different cells in each condition are shown. (**b**) Bar graphs (mean ± S.E.M) showing current densities at −100 mV holding potential under each condition. The statistical comparison among the values for all conditions was determined using one way ANOVA followed by Dunnett’s test.

To support our findings from the Ca^2+^ imaging experiments, we also carried out whole-cell recording of SOCE-representative current (*I*_CRAC_) from RBL-1H cells on a limited scale, following ~20 min pretreatment with the chosen drugs (20 μM) or BTP2 (10 μM). As shown in Fig. 3, teriflunomide and tolvaptan as well as BTP2 showed substantial and significant inhibition of the *I*_CRAC_. Other drugs showed similar trend but the results were not included due to inadequate number of experimental repeats.

**Figure 4:**
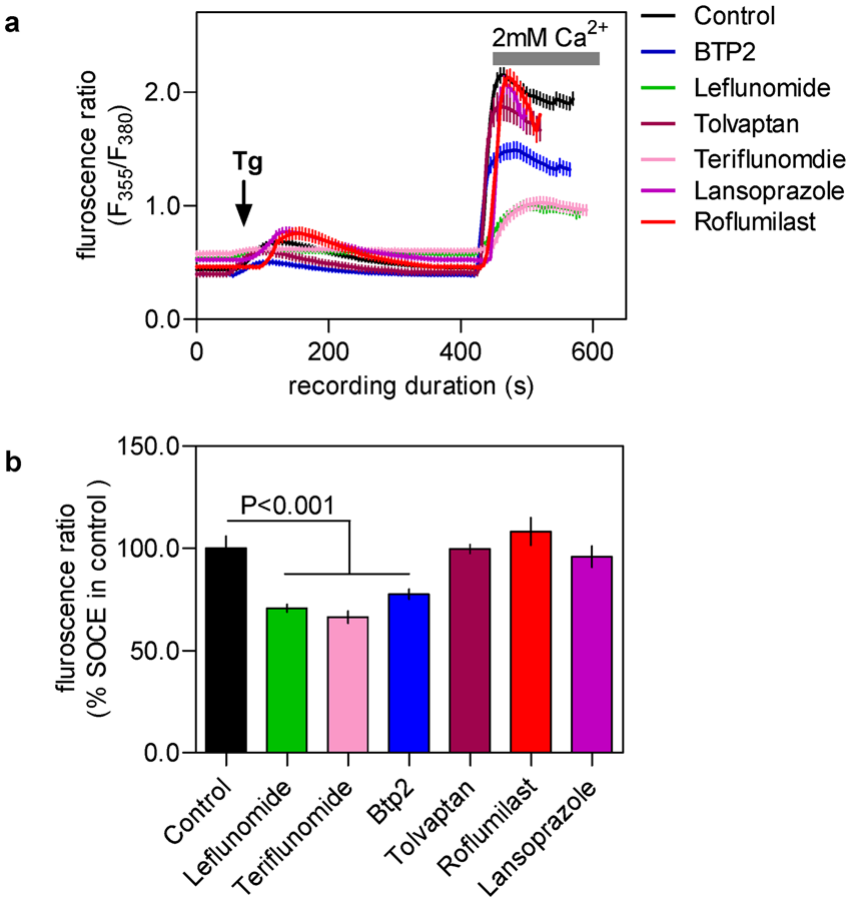
Effect of the chosen drugs on SOCE of RBL-1 cells following prolonged pre-treatment. Cells were treated with the indicated drugs at 1 μM dose for 24 hrs and then SOCE was triggered by thapsigargin and measured using standard protocol. (**a**) Sample traces of changes in Fura-2 fluorescence ratio representing Ca^2+^ signals following thapsigargin (2 μM) addition as well as Ca^2+^ add back under indicated conditions of pretreatment. (**b**) Histograms showing the peak SOCE levels triggered by Tg in control cells and cells pretreated with each drug at 1 μM dose for 24 hrs. Each value (mean ± SEM) was derived from 3–5 individual experiments and a total of 50–80 cells. The statistical comparison among the values for all conditions was determined using one way ANOVA followed by Dunnett’s test.

In some previous studies, pyrazole type inhibitors of SOCE such as BTP2 was found to inhibit SOCE more substantially following much longer (1 hr to 24 hr) pre-treatment^14,15^. All of our above mentioned drugs share reasonably good similarity in 3D shape and electrostatics with pyrazole type inhibitors such as BTP2 and Pyr6 (Supplementary Table 3, Supplementary Figs 3–6). We therefore sought to evaluate whether longer incubation of the cells with these drugs could improve their potency for SOCE suppression. For this, RBL-1H cells were pre-treated with 1 μM of the selected drugs and SOCE was recorded after 48 hours. As can be seen in Fig. 4, significantly inhibition of SOCE was observed only for cells treated with leflunomide, teriflunomide and BTP2.

In order to know whether the observed effect of the five drugs on SOCE was cell-type specific or not, we screened those 5 drugs at 10 μM dose against SOCE also in SHSY-5Y cells (a human neuroblastoma cell line) using the same protocol we used for the RBL-1 cells. As can be seen in Supplementary Fig. 7, all those drugs retained their ability to suppress SOCE significantly in SHSY-5Y cells as well, indicating that the observed effect was largely cell-type independent. It was interesting to note that the extent of SOCE suppression by all these drugs excluding roflumilast was seemingly higher for SHSY-5Y cells than in RBL-1 cells.

**Figure 5:**
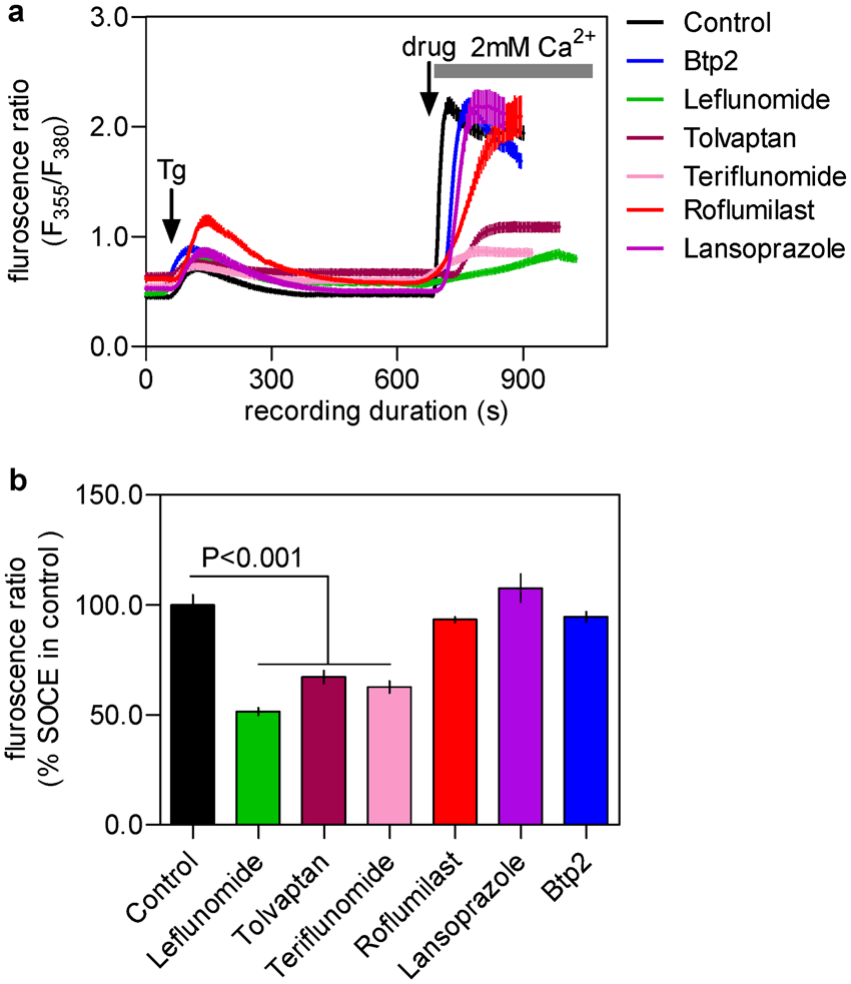
Effect of the chosen drugs given super-acutely on SOCE of RBL-1 cells. Indicated drugs at 50 μM dose were added to the extracellular solution ≤5 s (defined here as ‘super acute’ challenge) ahead of the Ca^2+^ addition during the Ca^2+^ imaging experiment using Fura-2. SOCE was triggered by thapsigargin and measured using standard protocol. (**a**) Sample traces of changes in Fura-2 fluorescence ratio representing Ca^2+^ signals following thapsigargin (2 μM) addition as well as Ca^2+^ add back under indicated conditions of drug pre-treatment. **(b)** Histograms showing the peak SOCE levels triggered by Tg in control cells and cells super-acutely exposed to each drug at 50 μM dose. Each value (mean ± SEM) was derived from 3–5 individual experiments and a total of 50–80 cells. The statistical comparison among the values for all conditions was determined using one way ANOVA followed by Dunnett’s test.

Hitherto we used our drugs in a ‘semi-acute’ manner i.e. we exposed the cells to the drugs ~20 min prior to the Ca^2+^ add back during the Ca^2+^ imaging experiments. Next we went on to evaluate whether the drugs could affect SOCE if added in a more acute way. With this aim, we applied the drugs at 50 μM concentration in a realistically acute manner i.e. ~10 s prior to adding Ca^2+^ to the extracellular media. It turned out that 3 drugs namely leflunomide, teriflunomide and tolvaptan, could significantly suppress SOCE when added in this acute fashion (Fig. 5). The known SOCE inhibitor - BTP2 as well as the other two drugs - roflumilast and lansoprazole were without any effect under such experimental condition.

**Figure 6:**
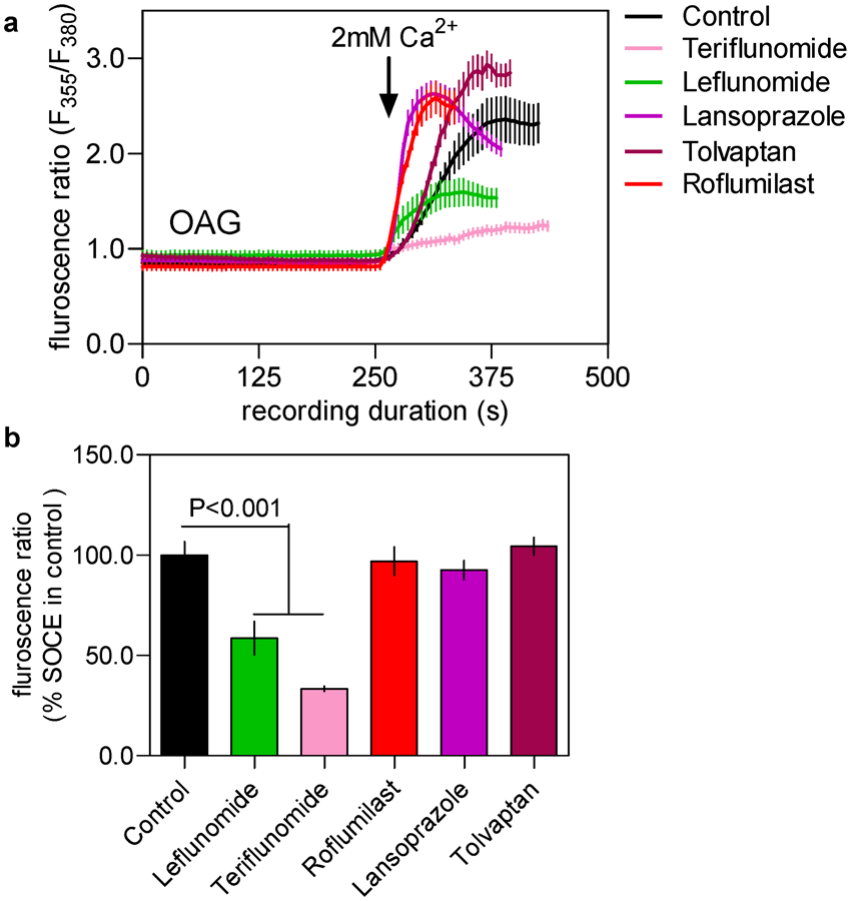
Effect of the chosen drugs on OAG-evoked Ca^2+^ entry in RBL-1 cells. (**a**) Sample traces representing Ca^2+^ signals (indicated by Fura-2 fluorescence ratio) triggered by adding 100 μM of OAG to RBL-1 cells with or without pretreatment of the chosen drugs (shown in various coloured lines, each at 10 μM dose). OAG is well known to be a selective activator of the members of the TRPC family selectively. (**b**) Histograms showing the peak OAG-activated Ca^2+^ entry in control cells and cells treated with 10 μM of each drug. Each value (mean ± SEM) was derived from 3–5 individual experiments and a total of 30–80 cells. The statistical comparison among the values for all conditions was determined using one way ANOVA followed by Dunnett’s test.

Next, we investigated whether the drugs that suppressed SOCE in our experiments, could affect Ca^2+^ entry in RBL-1H cells in response addition of 1-oleoyl-2-acetyl-sn-glycerol (OAG). The latter is a membrane permeable, synthetic analog of DAG and is well known to activate members of the TRPC family of ion channels including TRPC3, TRPC6 and TRPC7^16^. As can been seen in Fig. 6, addition of OAG triggered Ca^2+^ signals that were evident only in presence of extracellular Ca^2+^ in these cells, indicating that it was Ca^2+^ entry. Among the tested drugs at 10 μM dose, only leflunomide and teriflunomide showed significant inhibition of the OAG-evoked Ca^2+^ and thus these drugs are likely to have inhibitory effect on the activity of TRPC family members.

### Evaluation of the effects of chosen drugs on the nuclear translocation of NFAT

**Figure 7:**
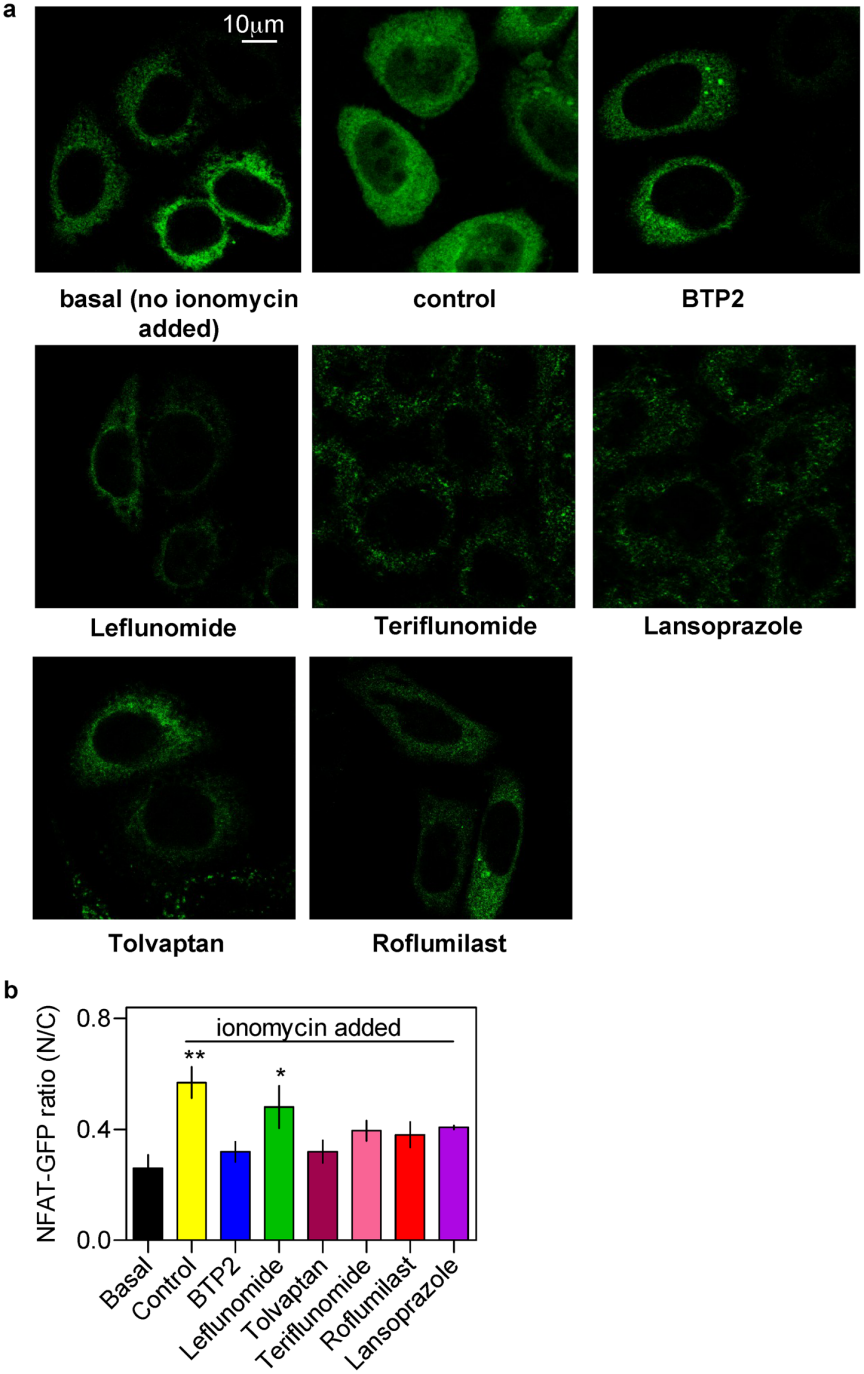
Effects of the chosen drugs and BTP2 on nuclear transolcation of NFAT. (**a**) Representative confocal images of HeLa cells showing subcellular localisation of NFAT (1–460)-GFP following application of ionomycin (1 μM for 40 min) preceded by pretreatment with the chosen drugs (20 μM) and BTP2 (10 μM) for 30 min. The basal and control groups received only DMSO pretreatment and no ionomycin was added to the basal group subsequently. (**b**) Bar graphs showing the nucleus to cytoplasm ratio (mean ± S.E.M.; n = 3) for the NFAT (1–460)-GFP under various conditions.

To determine whether the chosen drugs could affect SOCE-dependent cell signalling and relevant biological responses in intact cells, we evaluated their effect on the nuclear translocation of the nuclear factor of activated T cells (NFAT). The latter is a transcription factor which is well known in lymphocytes and relevant cells to require specifically the SOCE-derived Ca^2+^ signals for its activation^17^. For this, we used HeLa cells stably expressing NFAT1 (1-460)-GFP^18^. As can be observed in Fig. 7, ionomycin treatment (1 μM for 40 min) significantly increased the nuclear translocation of NFAT1 (1–460)-GFP in the control group but such increase was not observed for cells treated with BTP2 and all the chosen drugs except leflunomide. For the latter, visually no significant increment in the NFAT1 (1–460)-GFP fluorescence within the nuclear region was apparent but surprisingly the corresponding nucleus to cytoplasmic ratio was found to be significantly high when compared to the basal (i.e. non-ionomycin treated group).

### Evaluation of the effects of chosen drugs on the oligomerisation of STIM1 and Orai1

**Figure 8:**
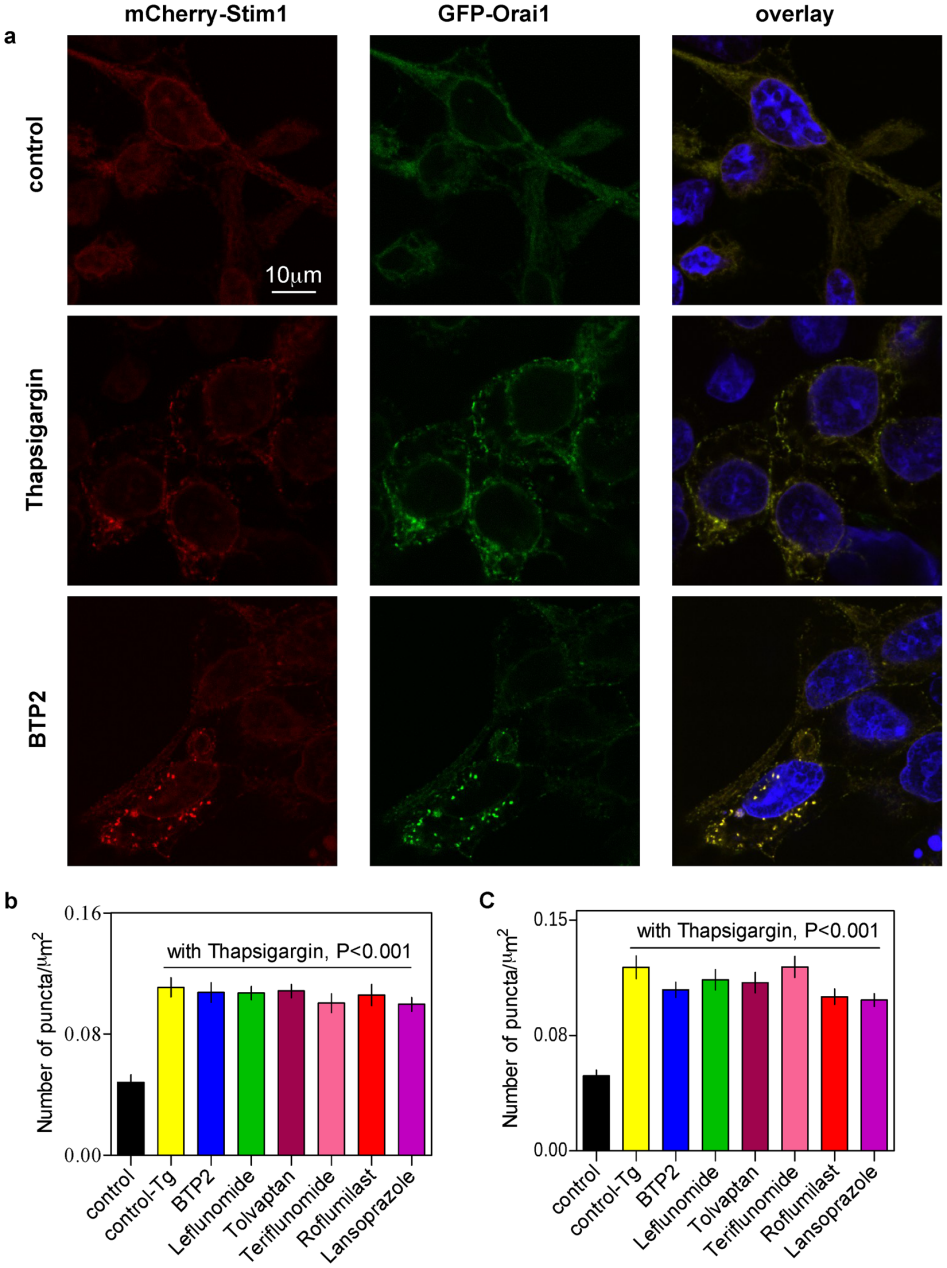
Effects of chosen drugs on clustering of STIM-1 and Orai-1 protein. (**a**) Representative fluorescent images of HEK293 cells coexpressing mCherry-STIM1 (red) and Orai1-eGFP (green) showing translocation and colocalization of both proteins in characteristic puncta after treatment with thapsigargin (Tg) (2 μM for 5 min). The scale bar is shown. GFP, green fluorescent protein. Quantification of mCherry-Stim1 (**b**) and GFP-Orai1 (**c**) puncta formation in response to Tg treatment and the effect of pre-treatment with drugs. The number of puncta formed in cells belonging to the control (no Tg/drug treatment) and the Tg-treated groups were determined using ImageJ software and were expressed as mean/μm^2^ of area ± SEM. Each bar represents data from 15–18 cells from 3 different experiments. The statistical comparison among the values for all conditions was determined using one way ANOVA followed by Dunnett’s test.

With a view to gaining some insight into the possible mechanism underlying the SOCE-inhibitor property of those 5 drugs, we evaluated the effects of the drugs on the clustering of STIM1 and also of Orai1, using confocal microscopy with a HEK293 cell line with an inducible mCherry-STIM1-T2A-Orai1-eGFP^19^. When assessed both qualitatively and quantitatively, we did not observe any significant change in the punctuate distribution of mCherry-STIM1 or Orai1-eGFP in presence and absence of any of the 5 drugs and BTP2, after cells were treated with 2 μM thapsigargin (Fig. 8, Supplementary Fig. 8). The results indicated that neither those drugs nor the known SOCE inhibitor, BTP2 affected STIM1 clustering or clustering of Orai1.

## Discussion

To date, there are several small molecule inhibitors of SOCE available and these notably include 2-APB (ant its analogs such as DPB162-AE and DPB163-AE), BTP2 (also known as YM-58483 or Pyr2), Pyr6, Synta 66 and AnCoA4^3–6^. Given the relative low potency and complex action of 2-APB on SOCE and on other ion channels^5^, we did not consider using 2-APB as bait. Since the other non-borinate SOCE inhibitors such as BTP2, Pyr6, Synta66 and AnCoA4 seem to have greater potency and selectivity, we used these agents as bait scaffolds (Supplementary Fig. 1) to scan conformer library made of drugs in clinical use. This led to identification of five drugs which suppressed SOCE significantly and dose-dependently in Ca^2+^ imaging using RBL-1 cells. The data was complemented by significant inhibition of *I*_CRAC_ by these drugs in whole-cell recordings from these cells. The drugs also retained their ability to inhibit SOCE in SHSY-5Y cells, indicating that the observed effect was not cell type specific. We also found that potency of two drugs – leflunomide and teriflunomide for inhibiting SOCE significantly improves upon much longer pre-incubation and these two drugs also could significantly inhibit Ca^2+^ signals mediated by TRPC channels.

It is well established that in lymphocyte and relevant cell lines including RBL cells, SOCE-derived Ca^2+^ signals are critically required for the calmodulin-dependent activation of the phosphatase calcineurin. The latter then dephosphorylates and thus promotes the nuclear translocation of NFAT isoforms. This pathway can be impaired by inhibition of SOCE which can be due to either structural defect in the underlying proteins (for e.g. R91W mutation in CRAC channel pore) or presence of SOCE blockers. In fact, nuclear translocation assay with a fluorescently-tagged NFAT provides for a useful a phenotypic screen to identify SOCE modulators or evaluate the consequences of perturbing SOCE in intact cells^2,3^. Given the observed SOCE-inhibitory property of the chosen drugs, it was satisfying to see their complementary ability to retard nuclear translocation of NFAT1(1–460)-GFP in HeLa cells. The only very odd exception to this was leflunomide, which qualitatively indicated similar trend with other drugs but upon quantitative assessment, was proven to be not affecting the nucleus to cytoplasm ratio of NFAT1(1–460)-GFP, significantly. Whether this is a genuine property of leflunomide stemming possibly from any off target effects, needs to be investigated further.

In our typical experiments, cells were exposed to the drugs ~20 min prior to the Ca^2+^ add back during the Ca^2+^ imaging experiments. During this time, the drugs presumably had opportunities for, among many other things, acting against STIM1 oligomerisation and/or STIM1-Orai1 coupling that are critical for SOCE activation. We therefore sought to know whether the drugs could affect SOCE upon more acute challenge, which could at least indicate whether they could inhibit SOCE when Orai1 is already activated, presumably through interaction with aggregated STIM1 proteins. It turned out that leflunomide, teriflunomide and tolvaptan when added more acutely to the bath solution, retained their ability to inhibit SOCE. The known SOCE inhibitor - BTP2 as well as the other two drugs - roflumilast and lansoprazole were without any effect under such experimental condition. Previously, Ishikawa *et al*.^20^ reported BTP2 to be equally efficacious as SOCE inhibitor in Jurkat T cells regardless of instantaneous or prolonged treatment. However, in another study^15^, substantial SOCE inhibition by BTP2 was evident in Jurkat T cells only when the cells were pre-incubated with the agent for no less than 2 hrs. He *et al*.^14^, on the other hand, found BTP2 to block SOCE in HEK-293 cells, DT40 cells and A7r5 cells within 10 min of application. Thus variability in the degree of SOCE inhibition by BTP2 and related molecules seem to be plausible depending on cell types and/or duration of treatment. The reason for such variation remains still unclear.

The fact that leflunomide, teriflunomide and tolvaptan could inhibit SOCE substantially even at acute challenge is intriguing. This indicates that these drugs could act on Orai1 channels that are activated already presumably via interaction with aggregated STIM1 proteins. In other possibilities, these drugs may act extracellularly or they may be readily cell-permeable if they act intracellularly. Although in acute challenge roflumilast and lansoprazole did not inhibit SOCE, it is difficult to be definitive about their lack of effect on STIM1 oligomerisation and/or coupling of STIM1-Orai1. This is because the known SOCE inhibitor - BTP2 which had no effect on STIM1 puncta formation^5^, was also ineffective in our acute challenge experiment. We therefore sought out to assess directly the effects of the drugs on the clustering of STIM1 and also of Orai1, using confocal microscopy with a HEK293 cell line with an inducible mCherry-STIM1-T2A-Orai1-eGFP^19^. All the five drugs as well as the known SOCE inhibitor, BTP2 did not affect STIM1 clustering or clustering of Orai1. In previous studies, the pyrazole derivatives such as BTP2, Pyr6, GSK7975A, GSK5503A etc. did not affect STIM1 puncta formaton and thus their SOCE-inhibitory properties were attributed to their possible interaction with the Orai1 proteins^5,21^. However, AnCoA4 is so far only the SOCE inhibitor for which the target - Orai1 has been experimentally validated^19^. Given the significant similarities of the drugs with the baits used in our study (Supplementary Table 3, Supplementary Figs 3–6) and given the observation that they do not interfere with STIM1 puncta formation (Fig. 8, Supplementary Fig. 8) in particular, we hypothesize that these agents inhibit SOCE probably via interacting with Orai1. This requires direct experimental validation in future.

It is interesting to note that leflunomide ranked the top (or nearly the top) in both ROCS and EON-based ranks of the hits obtained from screening with BTP2, Synta66 and Pyr6 as baits. Teriflunomide was also among the hits that ranked high in the lists, especially after re-ranking with EON (Supplementary Table 3). The EON-based electrostatic surface representations indicate that the isoxazole ring of leflunomide and the acrylonitrile moiety of teriflunomide share some electrostatic similarity with the o-fluoropyridyl group of Synta66 as well as the 4-methyl-1,2,3-thiadiazole-5-carboxamide moiety of BTP2. Also the fluoro-phenyl group of leflunomide and teriflunomide share some similarity with the 3,5-bis (trifluoromethyl) pyrazole moiety of BTP2 (Supplementary Fig. 3). Leflunomide is a pro-drug and ~70% of the orally-administered leflunomide rapidly undergoes opening of the isoxazole ring to produce the active malononitrilamide metabolite - teriflunomide^22^ (Supplementary Fig. 3). Both these agents have been clinically used as orally-active, disease-modifying immunosuppressive drugs for treating inflammatory diseases, often of autoimmune type. Leflunomide (Arava®) was approved and licensed in 1998 for the treatment of rheumatoid arthritis and psoriatic arthritis, whereas teriflunomide (Aubagio®) was synthesized later, approved and licensed in 2012 for the treatment of multiple sclerosis. Hitherto the only mechanism known for their immunosuppressive activity involves teriflunomide-mediated non-competitive, reversible inhibition of the mitochondrial enzyme dihydroorotate dehydrogenase (DHODH) - an enzyme expressed at high levels in proliferating lymphocytes. This results in blockade of *de novo* pyrimidine biosynthesis and therefore, reduced proliferation of activated lymphocytes^22^. However, there are some other cellular effects due to teriflunomide intake which cannot be explained by DHODH inhibition solely. These include impairment of cytokine production and expression of cell-surface molecules as well as cellular migration^23^. Thus it is fair to state that teriflunomide has immunological effects outside of its ability to inhibit pyrimidine synthesis in rapidly proliferating lymphocytes. So far it is known that higher doses of leflunomide and, consequently, of teriflunomide can inhibit few other targets including protein tyrosine kinases, cyclooxygenase-2, PDK1, NFκB, integrins etc^22^.

The average steady-state maximum plasma concentrations (C_max_) of teriflunomide achieved with doses used in clinical trials are 72 μM (7 mg) and 168 μM (14 mg)^24^. Thus based on our findings, teriflunomide and its prodrug leflunomide at clinically-relevant doses should have substantial inhibitory effect on SOCE. We propose that such effect should serve as an additional mechanism that significantly contributes to the observed biological effects and therapeutic benefits of these immunosuppressive drugs. Inhibition of SOCE can easily be correlated with decreased production of chemokines, decreased migration and proliferation lymphocytes, as observed for teriflunomide and/or leflunomide. That latter is indicated for rheumatoid arthritis and few recent studies have shown that SOCE inhibition can be an attractive strategy in treating such autoimmune diseases^25–27^.

Tolvaptan and conivaptan are orally and intravenously effective nonpeptide antagonists for the arginine vasopressin (AVP) receptor subtypes. These drugs are categorized as aquaretics i.e. they promote excretion of water without the loss of electrolytes and as such have been useful in treating hyponatremia^28,29^. In our ligand-based screening using BTP2, Synta66 and Pyr6, tolvaptan was within the top 20 of the hits ranked on the basis of ROCS and EON-based analysis. Conivaptan was within top 100 in ROCS and EON-based analysis of hits from Pyr6 and Synta66-based screening (Supplementary Table 3). Visually there were some similarities between the vaptans and the three individual baits (Supplementary Fig. 4). When screened in Ca^2+^ imaging at 10 μM dose (Fig. 1c), tolvaptan could still inhibit SOCE significantly whilst conivaptan was active only at 100 μM dose (Fig. 1b). Tolvaptan at therapeutic doses is however unlikely to affect SOCE as the C_max_ following a single 60 mg oral dose of tolvaptan to human subjects with normal kidney function was sub-micromolar^28,30^.

Omeprazole and lansoprazole (Supplementary Fig. 5) are gastric proton pump inhibitors and as such are widely used in treating peptic ulcer as well as few other gastroesophageal diseases including *Helicobacter pylori* infection^31^. Both these agents were within top 10 hits based on EON-ranking of hits derived from AnCoA4-based screening. Besides, omeprazole in particular also was within the top 50 hits based on EON-ranking of hits derived from screening with the other three baits (Supplementary Table 3). Qualitatively, these two drugs seem to share some similarities in surface electrostatics with the baits (Supplementary Fig. 5). Although omeprazole looked better in computational analysis (Supplementary Table 3), lansoprazole was more potent in inhibiting SOCE (Fig. 1c). However, the C_max_ of lansoprazole and omeprazole at clinically relevant oral doses (15–30 mg) is likely to be ≤ 3.5 μM^31^ and thus little effect on SOCE is predicted for this drug during clinical uses.

Roflumilast is an orally-active, selective inhibitor phosphodiesterase isoform 4 (PDE4) and as such it is approved for treating inflammatory conditions of the lungs such as chronic obstructive pulmonary disease (COPD). Selective inhibition of PDE4 by roflumilast has largely been attributed to its active metabolite – roflumilast N oxide. We included roflumilast in our final list of drugs for wet testing mainly because of the fact that this drug ranked second in both BTP2 as well as Pyr6-based screening using ROCS. Such high placement in the ranking seems to be due to significant similarity in 3D shape rather than in surface electrostatics (Supplementary Fig. 6). Besides, it was also among the top 55 hits in ROCS as well as EON-based ranking using AnCoA4 as the bait (Supplementary Table 3). At therapeutic doses (usually 500 μg once a day), the C_max_ of roflumilast N-oxide remains within low nanomolar range^32^, and therefore little effect on SOCE is anticipated.

## Conclusions

In summary, we have used 3D shape and electrostatics-based *in silico* screening to identify drugs that can inhibit SOCE dose-dependently, with no such prior records. It is noteworthy that two hits namely leflunomide and teriflunomide, could suppress SOCE significantly at therapeutically-achievable concentrations and this can potentially contribute to their observed immunosuppressive and other cellular effects. For the other three drugs namely lansoprazole, tolvaptan and roflumilast, the concentrations required to inhibit SOCE significantly are higher than cognate therapeutically-achievable maximum concentrations. They however, were more selective towards SOCE, unlike leflunomide and teriflunomide and thus can potentially serve as leads for future development of specific SOCE inhibitors.

## Methods

### Virtual screening, hit ranking and selection

We used the latest version of the eDrugs3D database (updated in July 2016) that contains 1822 molecular structures comprising of 3D representations along with isomers of total 1557 FDA-approved drugs^10^. For each drug, up to 100 conformers were generated using OMEGA^33^ (version 2.5.1.4, OpenEye Scientific Software, Santa Fe, NM, USA). The query molecules (SOCE inhibitors) such as BTP2, Synta66^3^, Pyr6^34^ and AnCoA4^19^ were drawn and energy-minimized with the Merck Molecular Force Field 94 using the ChemBioOffice Ultra 11.0 (CambridgeSoft Corporation, Cambridge, MA, USA). The energy-minimized structures of the query molecules were then used for generating the 3D queries (Supplementary Fig. 1). Each of the latter was then utilized to scan the above mentioned conformer library generated from eDrugs3D by using Rapid Overlay of Chemical Screens (ROCS)^35^ (version 3.2.1.4, OpenEye Scientific Software, Santa Fe, NM) and all resulting hits were ranked with Tanimoto Combo (TC) score that represents a numerical sum of the shape Tanimoto (maximum value = 1) and scaled colour Tanimoto score (maximum value = 1). We did not impose any cut off value for the TC score but limited the size of the screening output to 500 molecules. The ROCS output was then used as an input to EON (version 2.2.0.5, OpenEye Scientific Software, Santa Fe, NM) for re-ranking the aligned molecules on the basis of their electrostatic similarity with the cognate query ligand. For this, the EON Tanimoto combo (ET combo) score was used which is the sum of the EON Poisson-Boltzmann and EON Shape Tanimoto scores. Default parameters were used throughout. VIDA (version 4.3.0.4, OpenEye Scientific Software, Santa Fe, NM) was used to visualize and analyze the outputs from screening done with ROCS and EON. VIDA and MacPyMol (version 1.7.2.1) were used for molecular representations.

For each query molecule, we visually inspected each hit molecule using VIDA and shortlisted 11 candidates on the basis of several aspects including their similarity in electrostatic isopotential contour surfaces (EON-generated) with the cognate bait, their TC score and ET_pb score, their rank and frequency of appearing within the top 100 hits in ROCS as well as EON based screening, previous reported activity on calcium signalling and availability. The chosen molecules were purchased for bioassay. The drugs were dissolved in Ca^2+^-free Hank’s balanced salt solution (HBSS) or 10% DMSO.

All computational works were done in a Dell™ Precision Tower 7810 workstation (Intel® Xeon® Processor E5-1650 V3, 6 core, 3.5 GHz, 32GB RAM) run with 64 bit Windows 7 Professional as the operating system.

### Materials

Leflunomide, teriflunomide, tolvaptan and BTP2 were purchased from Tocris Bioscience (Bristol, UK). Omeprazole, lansoprazole, rufinamide, prazosin hydrochloride, terazosin hydrochloride, flutamide, roflumilast and propidium iodide were purchased from Sigma-Aldrich (Dorset, UK). Conivaptan hydrochloride was purchased from Selleckchem (distributed by Stratech, Suffolk, UK) and OAG was purchased from Cayman Chemicals (distributed by Cambridge Bioscience, Cambridge, UK). All molecules were of ≥98% purity certified by the vendors.

### Cell culture and media

RBL-1 cells were kindly provided by Professor Anant Parekh (Department of Physiology, Anatomy and Genetics, University of Oxford) and grown in RPMI 1640 medium supplemented with 10% fetal calf serum and maintained at 37 °C in 5% CO_2_ as mentioned previously^36^. SH-SY5Y cells were kindly provided by Professor Robin Irvine (Department of Pharmacology, University of Cambridge) and cultured in high glucose DMEM medium with the F-12 nutrients mixture, supplemented with 10% (v/v) fetal bovine serum, 2 mM L-glutamine and 1% (v/v) antibiotic/antimycotic mixture in humidified atmosphere with 5% CO2 and 95% air at 37 °C. A HEK293 cell line with an inducible mCherry-STIM1-T2A-Orai1-eGFP was kindly provided by Dr. Chan Young Park (School of Life Sciences, UNIST, South Korea) and was grown and maintained as per published report^19^. Cells were kept at 75 cm2 flask and passaged at least once in a week after reaching ~90% confluence. SH-SY5Y cells and RBL-1 Cells were not used beyond passage 25 and 16 respectively. In preparation for the passage of the cells, DMEM and trypsin media were pre-warmed at 37 °C. Cell medium from the flask was aspirated and cells were detached from the flask through addition of 2/3 ml of trypsin. After 2/3 min, the flasks were gently shaken to allow complete detachment of adhered cells and then trypsin was neutralized by adding 8–10 ml of DMEM. The cell suspension was then gently pipetted few times before it was centrifuged at 800 rpm for 5 minutes after which the supernatant was discarded and the cell pellet was re-suspended in DMEM (10 ml). The resultant cell suspension was used for seeding of cells into flasks and onto glass bottom dishes (10 mm) as required for Ca^2+^ signal recording.

HeLa cells stably expressing the NFAT1(1–460)-GFP fusion protein were a kind gift from Professor Patrick Hogan (La Jolla Instiute for Allergy and Immunology, CA, USA) and grown as per published protocol^18^.

### Measurement of intercellular Ca^2+^

Intracellular Ca^2+^ levels were measured by ratiometric imaging using fura-2 largely in accordance with previously published protocol^12^. Ratio fluorescence images were captured using a QIClick™ digital CCD camera (QImaging, BC, Canada) mounted on a Nikon Eclipse Ti-S Microscope. Consecutive excitation was provided by a Dual OptoLED Power Supply (Cairn), alternating between both 355 nm (*F*_355_) and 380 nm (*F*_380_) wavelength LEDs. Emission fluorescence was collected at 510 nm (470 nm–550 nm). 12-bit images were acquired in every 5 seconds with MetaFluor® (Molecular Devices, USA) Fluorescence Ratio Imaging Software. The fluorescence at each time-point was extracted for both 355 nm and 380 nm wavelengths, corrected for autofluorescence and the 355 nm/380 nm ratios (*F*_355_/*F*_380_) were then calculated to represent intracellular Ca^2+^ levels. All Ca^2+^ imaging experiments were done in room temperature.

### Confocal Microscopy

Cells were plated onto sterilized cover slips coated with poly-D-lysine and maintained in complete DMEM. STIM1-mCherry and Orai1-GFP protein were induced through tetracycline (2–5 μM) treatment as per published protocol^19^, 12–16 hours before the experiment. Cells were washed with HBSS and incubated for 10–15 minute with the respective drug followed by 2 μM of TG treatment for 5 min. Cells were fixed with ice cold methanol (100%).

Confocal images were acquired using a Leica SP5 microscope equipped with a Plan Apochromat 63x/NA1.4 oil immersion objective. The excitation wavelengths (ex) and emission filters (em) were as follows: DAPI, ex 364 nm/em 385–470 nm; Orai1-GFP or NFAT1(1–460)-GFP, ex 488 nm/em 500–515 nm; STIM1-mCherry, ex 543 nm/em 610 nm. Acquired images were processed using the ImageJ (National Institutes of Health). For quantification of STIM1–mCherry and Orai1-GFP puncta formation, the puncta were selected as spots of high fluorescence intensity ranging from approx. 0.5 to 1.0 μm in diameter and their number was determined as per previously published method^37^.

For the quantitative assessment of the ionomycin (1 μM for 40 min)-induced nuclear translocation of NFAT1(1–460)-GFP in HeLa cells with or without drug/BTP2 pretreatment, the method described by Kar *et al*.^17^ was followd. All images were prepared for publication using Adobe Photoshop.

### Evaluation of cytotoxicity

Cell death was assessed by propidium iodide staining as per the method previously published by Lecoeur^13^. Briefly, RBL-1 cells were first treated with drugs (the maximum concentration used in functional assay i.e. 300 µM), BTP2 (30 µM) or vehicle (2 µM DMSO). Afterwards, cells were incubated with 10 µg/ml propidium iodide for 20 min at 37 °C. Cells were then washed with PBS and fixed for 15 min with 100% ice cold methanol in −20 °C. Cells within each treatment group were later imaged using confocal microscopy with the 20x objective.

### Electrophysiology

CRAC currents (*I*_CRAC_) were recorded from RBL-1 cells at the room temperature (22–25 °C) in the tight-seal, whole-cell configuration as previously described^38^. Patch pipettes were pulled from borosilicate glass capillaries (World Precision Instruments, Hitchin, UK) and fire polished. Pipettes had resistances of 2.5–4 MΩ when filled with standard internal solution containing (in mM): cesium glutamate 145, NaCl 8, MgCl_2_ 1, MgATP 2, EGTA 10, HEPES 10, pH adjusted to 7.2 with CsOH. IP_3_ (30 μM) was also included in the pipette solution. Extracellular solution contained (in mM): NaCl 145, KCl 2.8, CaCl_2_ 10, MgCl_2_ 2, CsCl 10, glucose 10, HEPES 10, pH adjusted to 7.4 with NaOH. All voltages were corrected for a liquid junction potential of +10 mV between external and internal solutions. *I*_CRAC_ was measured using voltage ramps (−100 to +100 mV in 50 ms) applied every 2 s. Axopatch 200 B and Digidata 1440 A (Molecular Devices) with pCLAMP 10 software were used for data acquisition and analysis. Data were digitized at 10 kHz and filtered at 1 kHz. Cells were held at 0 mV between pulses. Capacitative currents were cancelled before each voltage ramp. Uncompensated series resistance was in the range of 5–14 MΩ. All leak currents were subtracted by averaging the first 2–3 ramp currents obtained just after the break in and then subtracting this from all subsequent currents. Amplitude of the current was normalized to cell size and expressed in pA/pF. Cells were pre-treated with BTP2 (10 μM)/ drugs (20 μM) for ~20 min prior to recording.

### Statistical analysis

Results were expressed as means ± SEM for n independent experiments, with each experiment performed minimally in triplicate. The latter in this study involved Ca^2+^ imaging of drug-treated cells in three different days (at least 3 petri-dish of cells per drug per day) done with paired control group (3 petri-dish of cells per day). One way ANOVA followed by a post hoc (Dunnett) test was to identify significant differences, unless specified otherwise. Statistical significance was set at P < 0.01 and all statistical analyses were done using GraphPad Prism 5 (GraphPad software Inc., CA, USA).

### Data availability statement

Most of the data generated or analyzed during this study are included in this article (and its Supplementary information files). Detailed lists of hits obtained with four different baits are available from the corresponding author on reasonable request.

## Electronic supplementary material

Supplementary Information

## References

[1] Berridge MJ, Lipp P, Bootman MD (2000). The versatility and universality of calcium signalling. Nature reviews. Molecular cell biology.

[2] Prakriya M, Lewis RS (2015). Store-Operated Calcium Channels. Physiol Rev.

[3] Parekh AB (2010). Store-operated CRAC channels: function in health and disease. Nat Rev Drug Discov.

[4] Tian C, Du L, Zhou Y, Li M (2016). Store-operated CRAC channel inhibitors: opportunities and challenges. Future Med Chem.

[5] Jairaman A, Prakriya M (2013). Molecular pharmacology of store-operated CRAC channels. Channels (Austin).

[6] Sweeney ZK, Minatti A, Button DC, Patrick S (2009). Small-molecule inhibitors of store-operated calcium entry. ChemMedChem.

[7] Pevarello P (2014). Ca^2+^ release-activated Ca^2+^ channel inhibitors. Pharmaceutical patent analyst.

[8] AbdulHameed MD (2012). Exploring polypharmacology using a ROCS-based target fishing approach. Journal of chemical information and modeling.

[9] Vasudevan SR, Moore JB, Schymura Y, Churchill GC (2012). Shape-based reprofiling of FDA-approved drugs for the H(1) histamine receptor. J Med Chem.

[10] Pihan E, Colliandre L, Guichou JF, Douguet D (2012). e-Drug3D: 3D structure collections dedicated to drug repurposing and fragment-based drug design. Bioinformatics.

[11] Bird GS, DeHaven WI, Smyth JT, Putney JW (2008). Methods for studying store-operated calcium entry. Methods.

[12] Hendron E (2014). Potent functional uncoupling between STIM1 and Orai1 by dimeric 2-aminodiphenyl borinate analogs. Cell calcium.

[13] Lecoeur H (2002). Nuclear apoptosis detection by flow cytometry: influence of endogenous endonucleases. Experimental cell research.

[14] He LP, Hewavitharana T, Soboloff J, Spassova MA, Gill DL (2005). A functional link between store-operated and TRPC channels revealed by the 3,5-bis(trifluoromethyl)pyrazole derivative, BTP2. J Biol Chem.

[15] Zitt C (2004). Potent inhibition of Ca2 + release-activated Ca2 + channels and T-lymphocyte activation by the pyrazole derivative BTP2. J Biol Chem.

[16] Hofmann T (1999). Direct activation of human TRPC6 and TRPC3 channels by diacylglycerol. Nature.

[17] Kar P, Nelson C, Parekh AB (2011). Selective activation of the transcription factor NFAT1 by calcium microdomains near Ca2 + release-activated Ca2 + (CRAC) channels. J Biol Chem.

[18] Gwack Y (2006). A genome-wide Drosophila RNAi screen identifies DYRK-family kinases as regulators of NFAT. Nature.

[19] Sadaghiani AM (2014). Identification of Orai1 channel inhibitors by using minimal functional domains to screen small molecule microarrays. Chem Biol.

[20] Ishikawa J (2003). A pyrazole derivative, YM-58483, potently inhibits store-operated sustained Ca^2+^ influx and IL-2 production in T lymphocytes. J Immunol.

[21] Derler I (2012). The action of selective CRAC channel blockers is affected by the Orai pore geometry. Cell calcium.

[22] Fragoso YD, Brooks JB (2015). Leflunomide and teriflunomide: altering the metabolism of pyrimidines for the treatment of autoimmune diseases. Expert review of clinical pharmacology.

[23] Korn T, Magnus T, Toyka K, Jung S (2004). Modulation of effector cell functions in experimental autoimmune encephalomyelitis by leflunomide–mechanisms independent of pyrimidine depletion. Journal of leukocyte biology.

[24] Oh J, O’Connor PW (2013). An update of teriflunomide for treatment of multiple sclerosis. Therapeutics and clinical risk management.

[25] Guo L (2014). A comparison of various optimization algorithms of protein-ligand docking programs by fitness accuracy. Journal of molecular modeling.

[26] Liu S, Kiyoi T, Takemasa E, Maeyama K (2017). Intra-articular lentivirus-mediated gene therapy targeting CRACM1 for the treatment of collagen-induced arthritis. Journal of pharmacological sciences.

[27] Liu S (2014). Upregulation of store-operated Ca(2+) entry in the naive CD4(+) T cells with aberrant cytokine releasing in active rheumatoid arthritis. Immunology and cell biology.

[28] Bhatt PR, McNeely EB, Lin TE, Adams KF, Patterson JH (2014). Review of Tolvaptan’s Pharmacokinetic and Pharmacodynamic Properties and Drug Interactions. Journal of clinical medicine.

[29] Aditya S, Rattan A (2012). *Vaptans: A new option in the manag*ement of hyponatremia. International journal of applied & basic medical research.

[30] Shoaf SE, Bricmont P, Mallikaarjun S (2014). Pharmacokinetics and pharmacodynamics of oral tolvaptan in patients with varying degrees of renal function. Kidney international.

[31] Shin JM, Kim N (2013). Pharmacokinetics and pharmacodynamics of the proton pump inhibitors. Journal of neurogastroenterology and motility.

[32] Rabe KF (2011). Update on roflumilast, a phosphodiesterase 4 inhibitor for the treatment of chronic obstructive pulmonary disease. British journal of pharmacology.

[33] Hawkins PC, Skillman AG, Warren GL, Ellingson BA, Stahl MT (2010). Conformer generation with OMEGA: algorithm and validation using high quality structures from the Protein Databank and Cambridge Structural Database. Journal of chemical information and modeling.

[34] Schleifer H (2012). Novel pyrazole compounds for pharmacological discrimination between receptor-operated and store-operated Ca(2+) entry pathways. British journal of pharmacology.

[35] Hawkins PC, Skillman AG, Nicholls A (2007). Comparison of shape-matching and docking as virtual screening tools. J Med Chem.

[36] Bianchi L (1996). Regulation by spermine of native inward rectifier K+ channels in RBL-1 cells. J Biol Chem.

[37] Yazbeck P (2017). STIM1 Phosphorylation at Y361 Recruits Orai1 to STIM1 Puncta and Induces Ca2+ Entry. Scientific reports.

[38] Glitsch MD, Bakowski D, Parekh AB (2002). Effects of inhibitors of the lipo-oxygenase family of enzymes on the store-operated calcium current I(CRAC) in rat basophilic leukaemia cells. The Journal of physiology.

