# Supplementary material for: Unveiling some FDA-approved drugs as inhibitors of the store-operated Ca2+ entry pathway
Source: Sci Rep. 2017 Oct 16;7:12881. doi: 10.1038/s41598-017-13343-x (PMC5643495; doi:10.1038/s41598-017-13343-x)
Supplement: Supplementary file 1 — Supplementary Information [file 41598_2017_13343_MOESM1_ESM.pdf]

Unveiling some FDA-approved drugs as inhibitors of the store-operated Ca^{2+} entry pathway

Saifur Rahman, Taufiq Rahman[*]

Department of Pharmacology, University of Cambridge, Tennis Court Road, Cambridge, UK

*Correspondence
Dr Taufiq Rahman, Department of Pharmacology, University of Cambridge, Tennis Court Road, CB2 1PD, UK. E-mail: mtur2@cam.ac.uk

Supplementary Figure 1
2D representations of the bait molecules and their corresponding 3D queries used in the ligand-based virtual screening. The 3D queries were generated using vROCS (version 3.2.1.4, OpenEye Scientific Software, Santa Fe, NM).

Supplementary Figure 2
Evaluation of cytotoxicity of the chosen drugs at their maximum concentration used in the study**. (a)** Typical images representing propidium iodide staining for the control cells as well as well cells exposed to 300μM of drugs for ~15min. Only BTP2 and teriflunomide group are shown as example. **(b)** Summary data showing the percentage of dead cells within each treatment group. Each histogram represents mean ± SEM and was derived from 3–5 individual experiments and a total of 30–80 cells. The statistical comparison among the values for all conditions was determined using one way ANOVA followed by Dunnett's test.

Supplementary Figure 3
Structures of leflunomide and teriflunomide and their apparent similarity with few known SOCE-inhibitors. **(a)** 2D representations of leflunomide and its active metabolite, teriflunomide. **(b)** 3D chemical structures of leflunomides, teriflunomides and the known SOCE-inhibitors namely BTP2, Synta66 and Pyr6 with electrostatic surfaces coded by color (red for negative and blue for positive).These surface electrostatic electrostatic representations were generated by EON from ROCS-based overlay of baits and hits during respective ligand-based virtual screening.

Supplementary Figure 4
Structures of tolvaptan and conivaptan and their apparent similarity with few known SOCE-inhibitors. **(a)** 2D representations of tolvaptan and conivaptan **(b)** 3D chemical structures of tolvaptan and conivaptan and the known SOCE-inhibitors namely BTP2, Synta66 and Pyr6 with electrostatic surfaces coded by color (red for negative and blue for positive).These surface electrostatic electrostatic representations were generated by EON from ROCS-based overlay of baits and hits during respective ligand-based virtual screening.

Supplementary Figure 5
Structures of omeprazole and lansoprazole and their apparent similarity with few known SOCE-inhibitors. **(a)** 2D representations of omeprazole and lansoprazole **(b)** Three-dimensional chemical structures of omeprazole and lansoprazole and the known SOCE-inhibitors namely BTP2, Pyr6, Synta66 and AnCoA4 with electrostatic surfaces coded by color (red for negative and blue for positive).These surface electrostatic electrostatic representations were generated by EON from ROCS-based overlay of baits and hits during respective ligand-based virtual screening.

Supplementary Figure 6
Comparison of 3D shape and surface electrostatics of roflumilast with some known SOCE-inhibitors. **(a)** 2D representations of roflumilast **(b)** 3D shapes of BTP2, Pyr6 and roflumilast. These shapes were generated by ROCS (OpenEye) (C) 3D chemical structures of roflumilast and the known SOCE inhibitors Pyr6 and AnCoA4 with electrostatic surfaces coded by colour (red for negative and blue for positive).These surface electrostatic electrostatic representations were generated by EON from the ROCS-based overlay of baits and hits during respective ligand-based virtual screening.

Supplementary Figure 7

Evaluation of the effects of chosen drugs on the SOCE in SHSY-5Y cells triggered by thapsigargin in Fura-2 based Ca^{2+} imaging experiments. **(a)** Sample traces representing Ca^{2+} signals (indicated by Fura-2 fluorescence ratio) triggered by adding 2μM of thapsigargin (Tg) to SHSY-5Y cells with or without pre-treatment of the chosen drugs (shown in various coloured lines, each at 10μM dose). **(b)** Histograms showing the peak SOCE levels triggered by Tg in control cells and cells pre-treated with each drug at 10μM dose**.** Each value (mean ± SEM) was derived from 3–5 individual experiments and a total of 30–80 cells. The statistical comparison among the values for all conditions was determined using one way ANOVA followed by Dunnett's test.

Supplementary Figure 8

Effects of chosen drugs on clustering of STIM-1 and Orai-1 protein. Representative fluorescent images of representative human embryonic kidney 293 (HEK293) cells coexpressing STIM1Cherry (red) and Orai1GFP (green) showing translocation and colocalization of both proteins in characteristic puncta after treatment with thapsigargin (Tg) (2μM for 5 min). The scale bar is shown.

Supplementary Figure 1

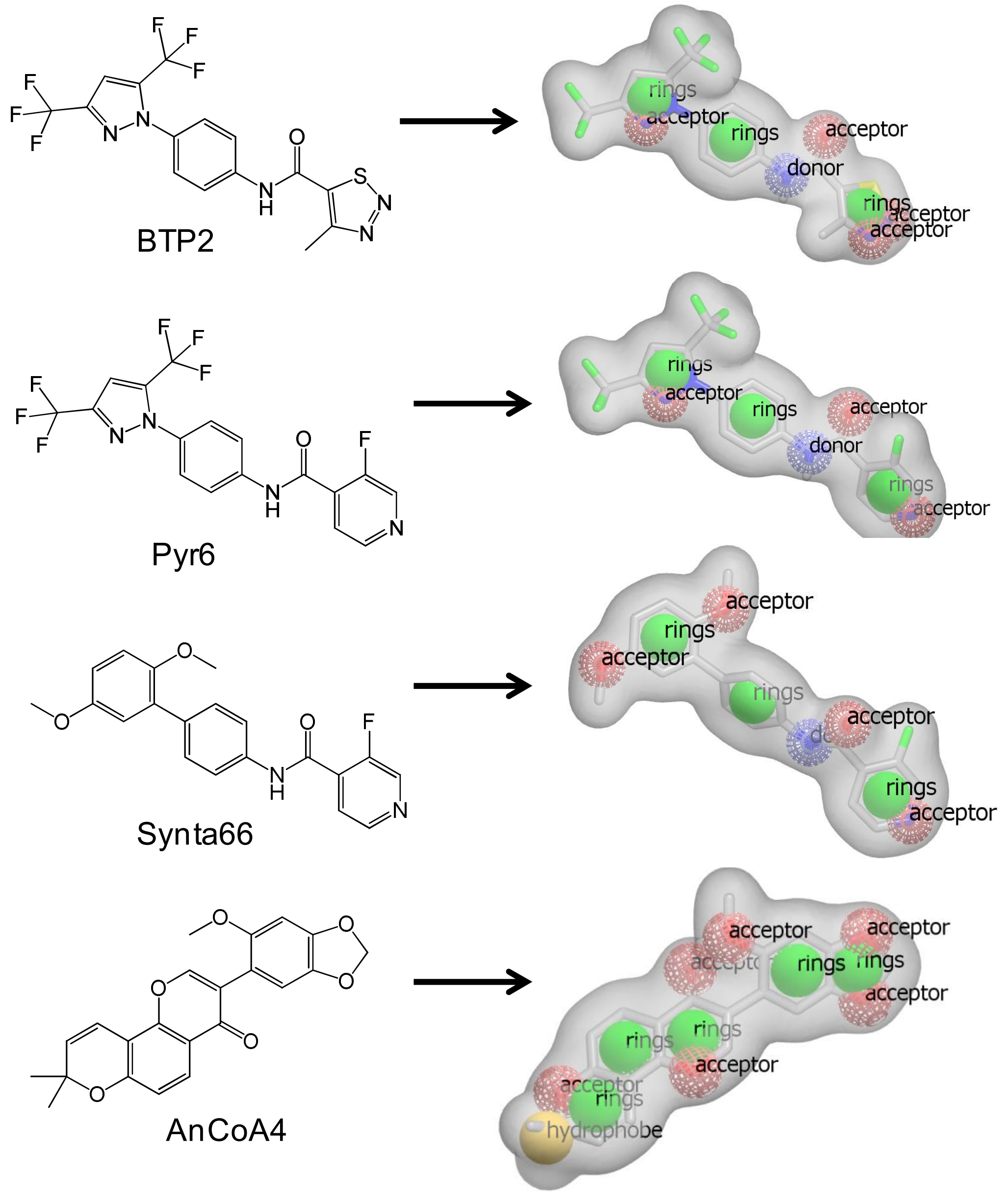

Supplementary Figure 2

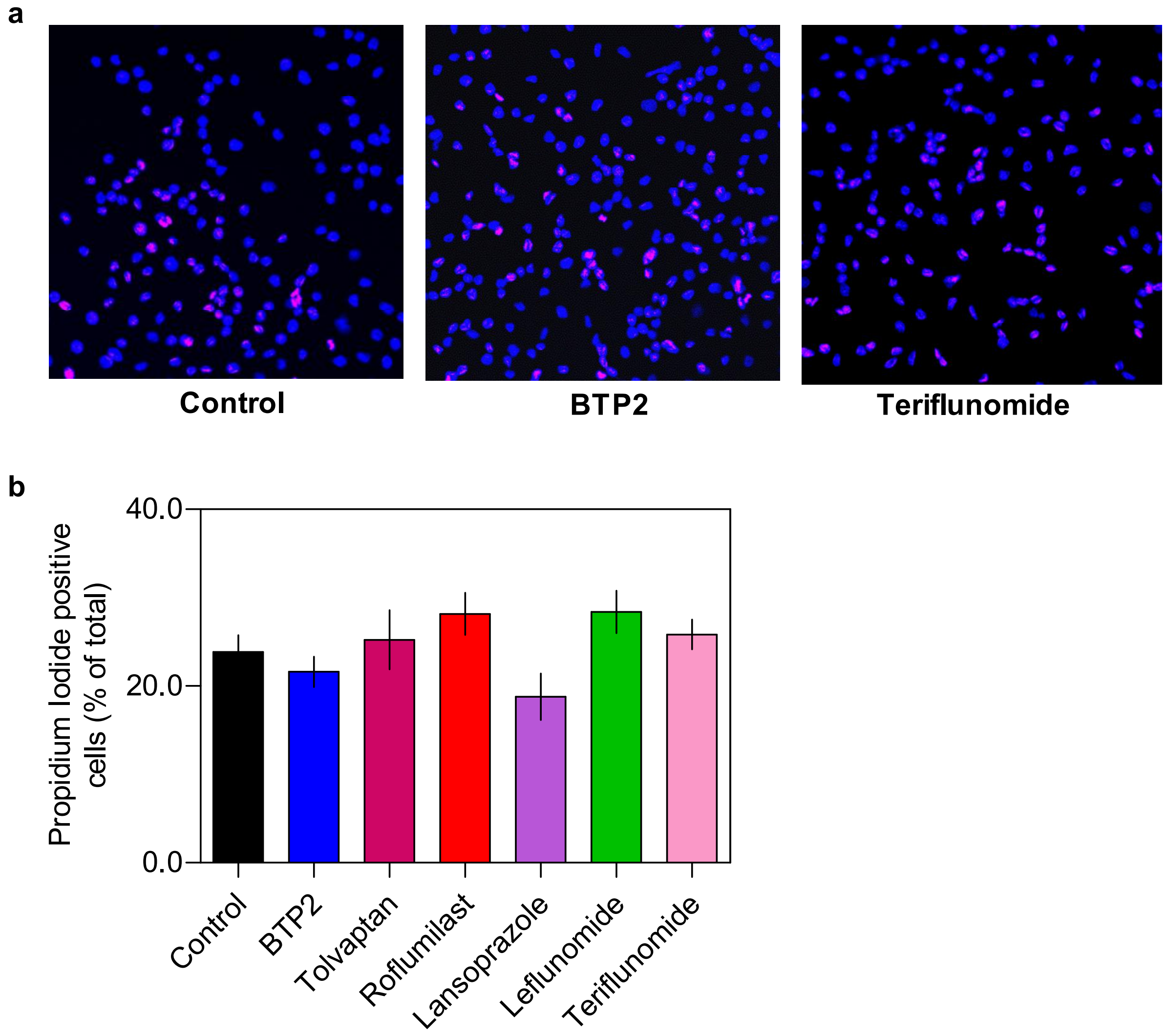

Supplementary Figure 3

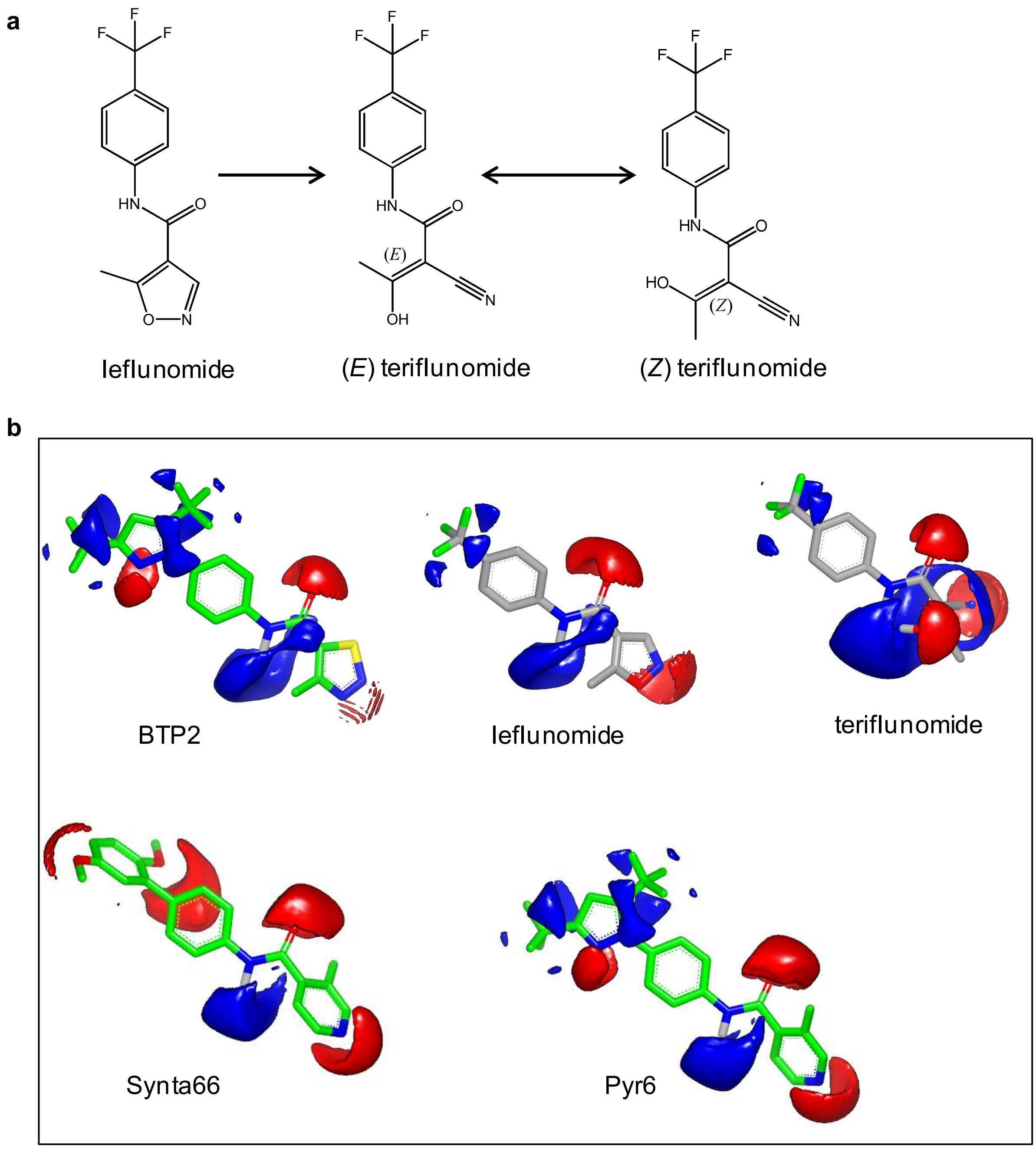

Supplementary Figure 4

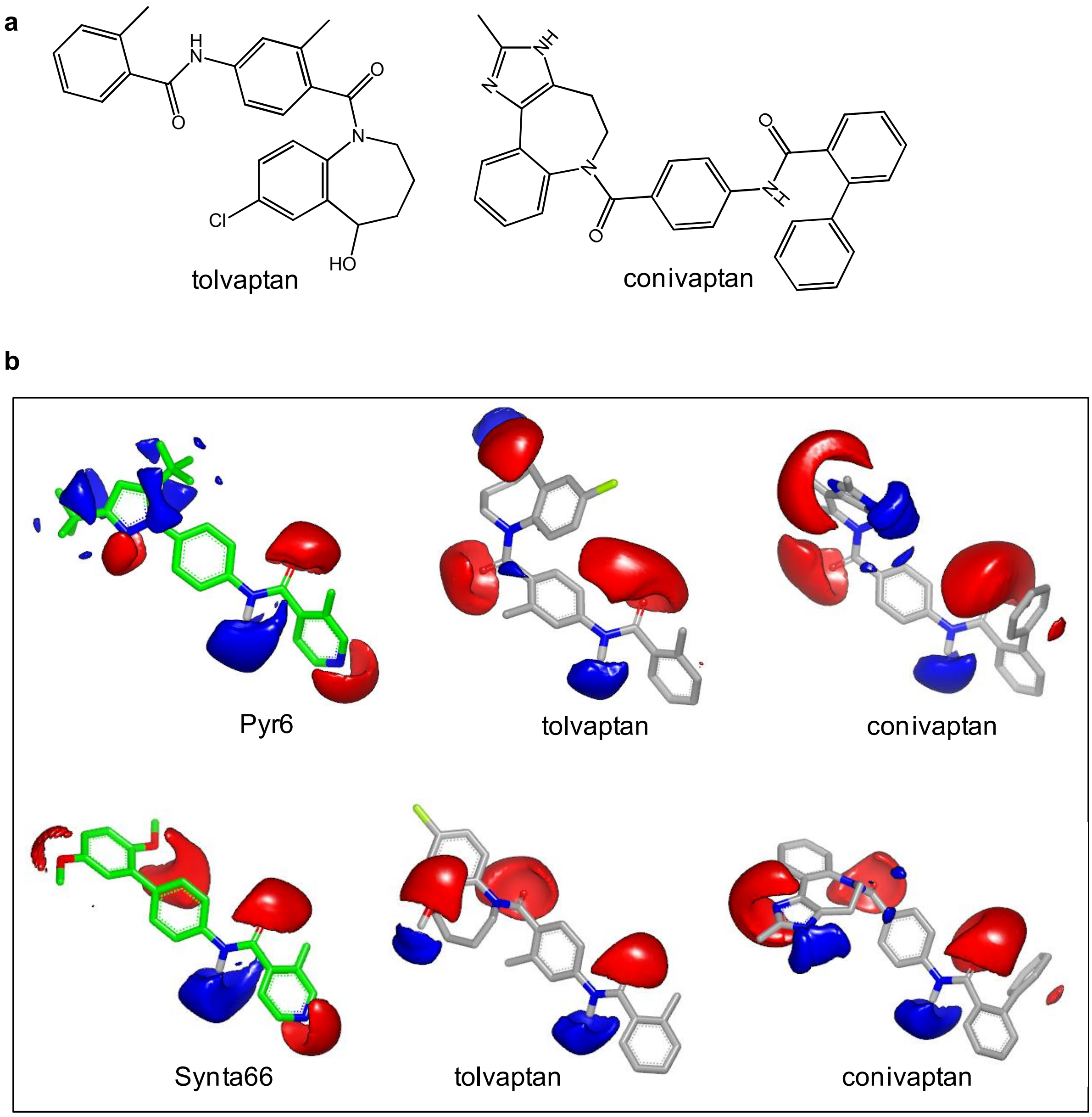

Supplementary Figure 5

a

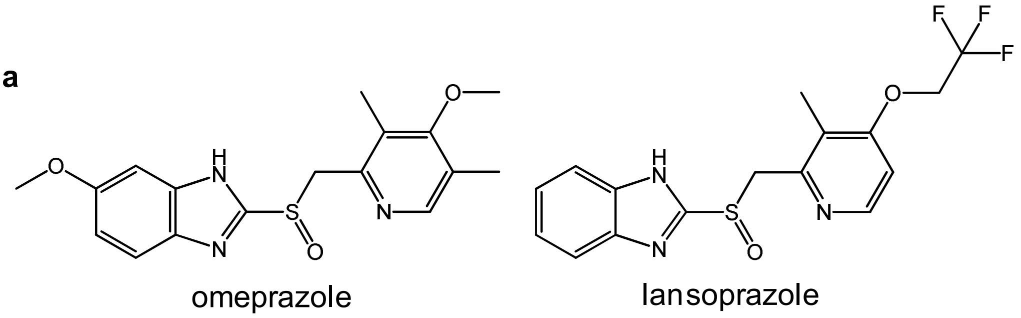

b

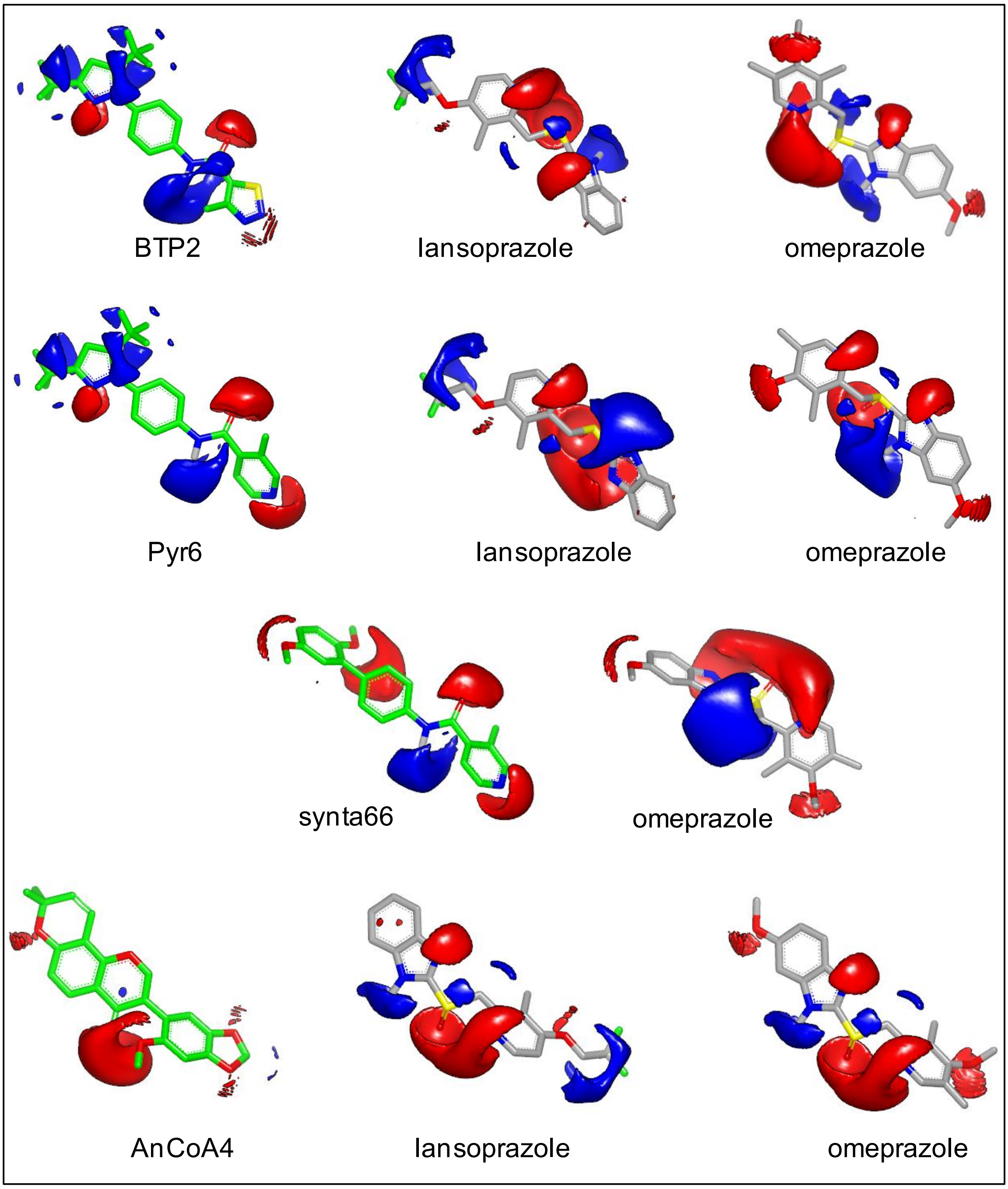

Supplementary Figure 6

a

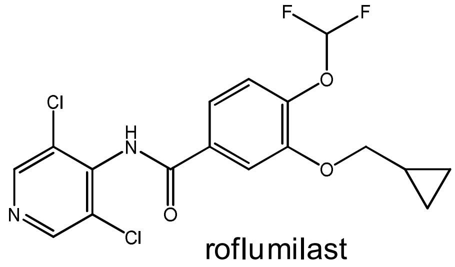

b

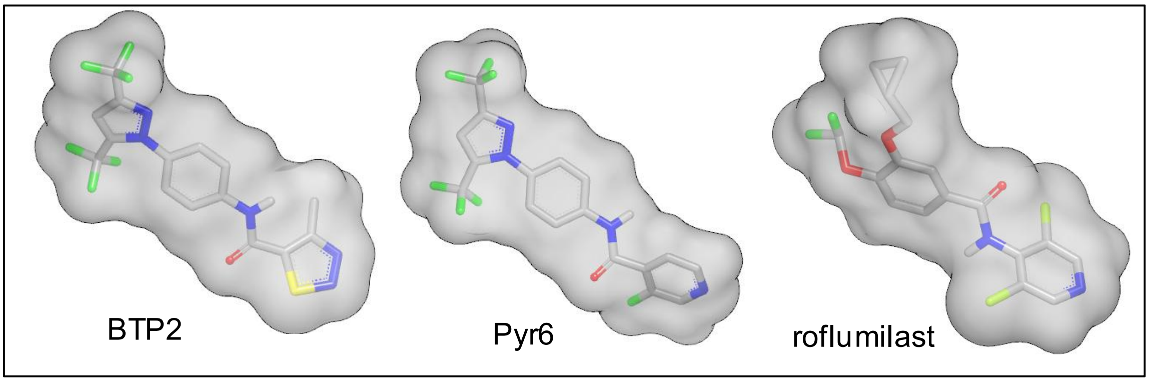

c

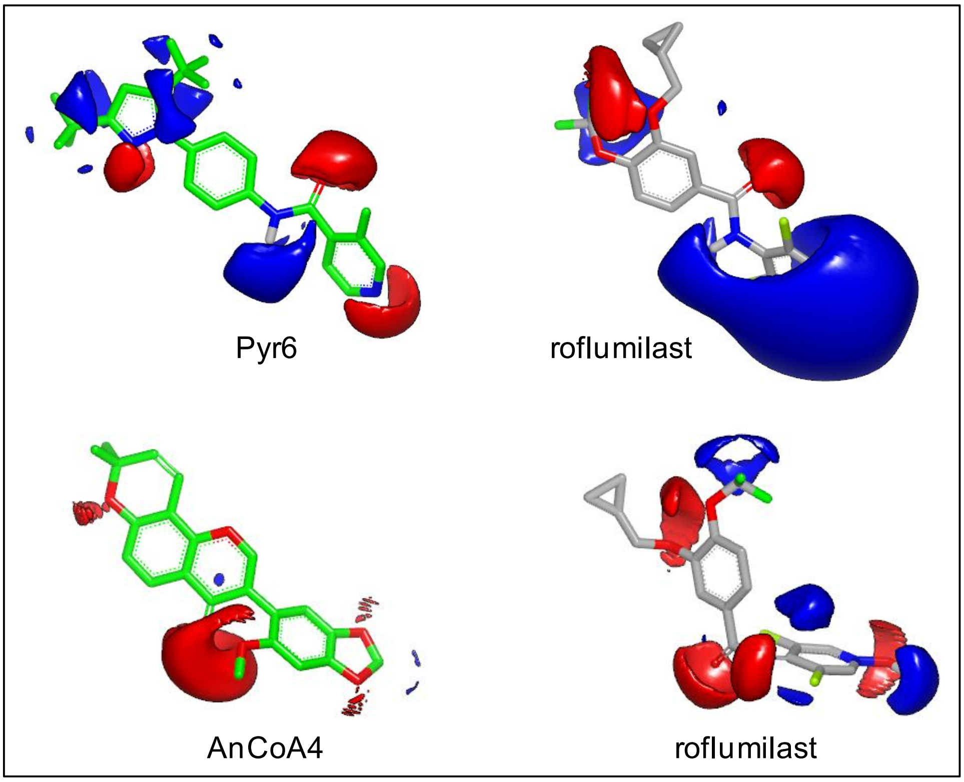

Supplementary Figure 7

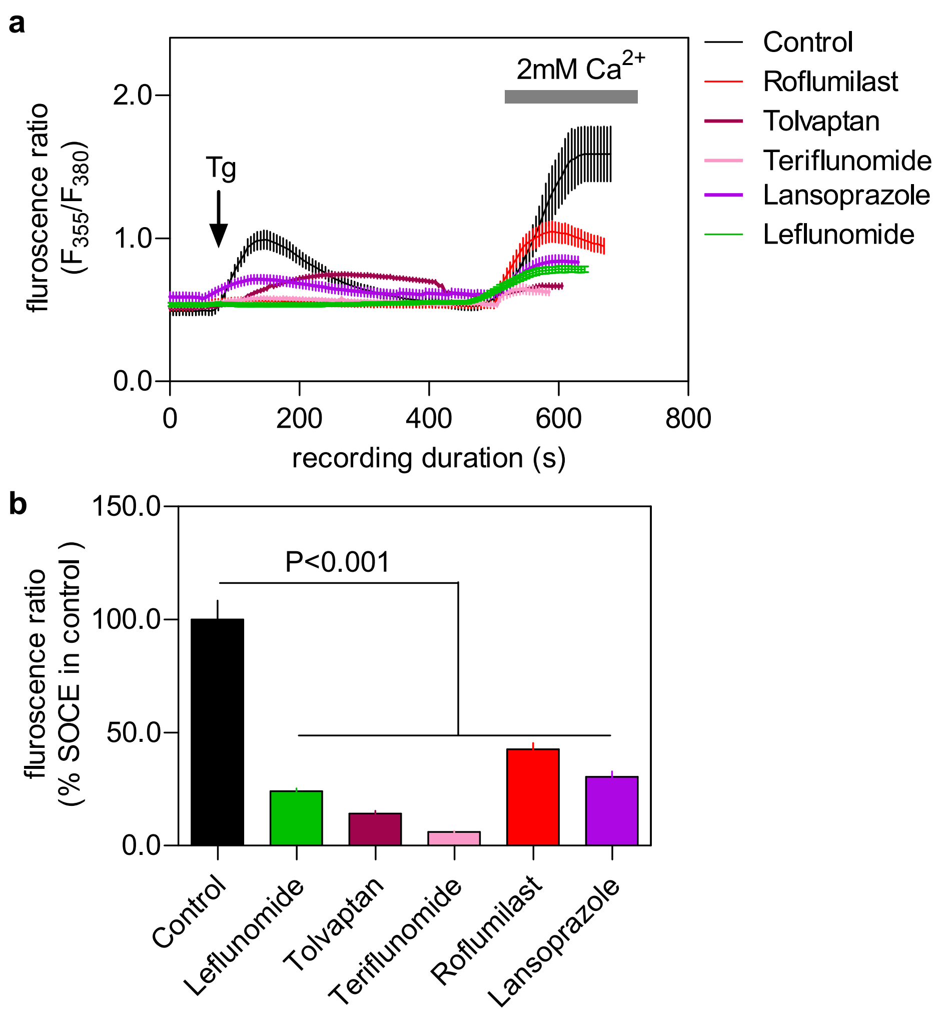

Supplementary Figure 8

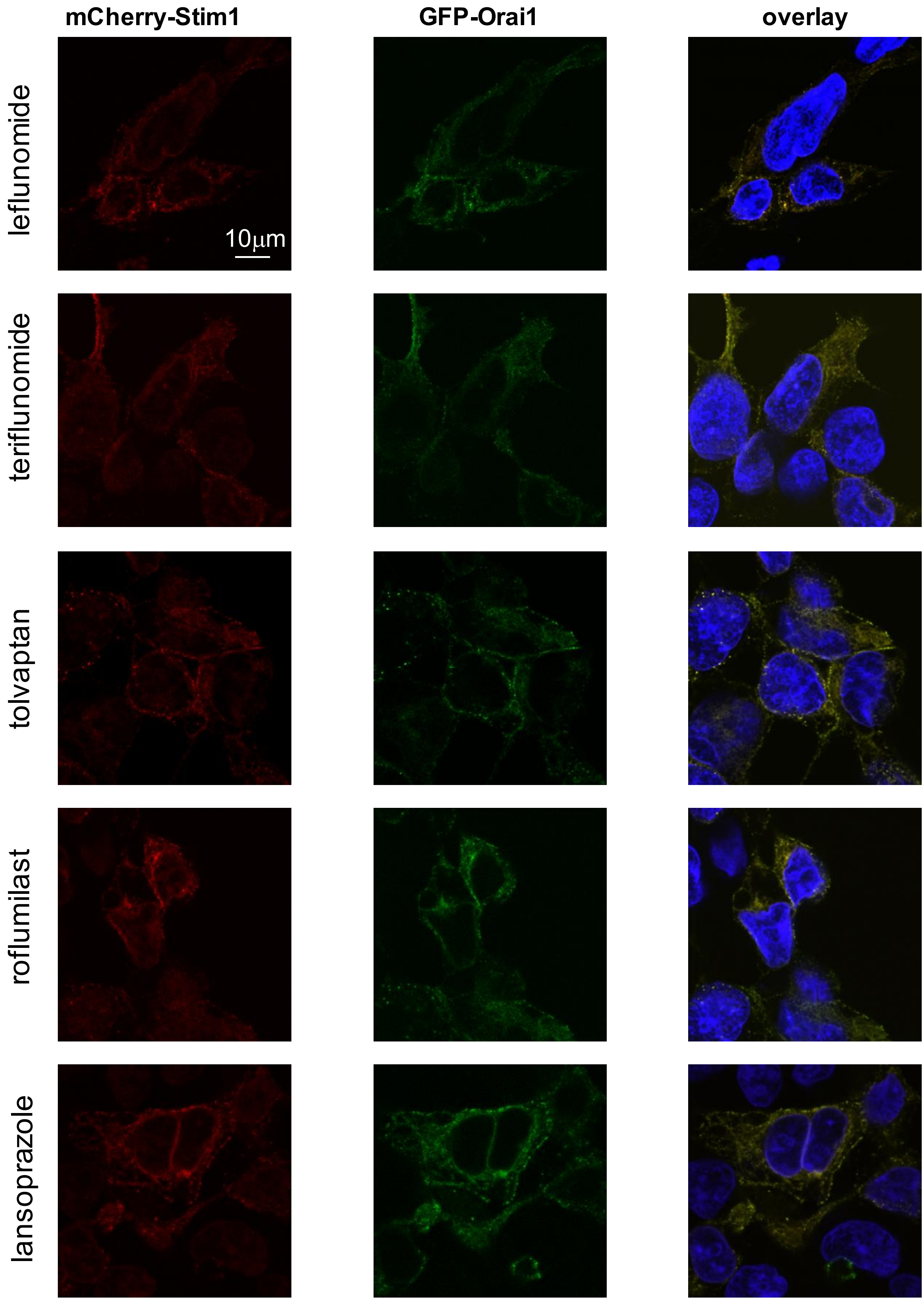

Supplementary Table 1

ROC and EON based ranking of hits from BTP2-basd screening of TOCRIS library

		ROC related				EON related		
	zinc_id	TanimotoCombo	ShapeTanimoto	ColorTanimoto	**ROC Rank**	EON_ET_pb	EON_ET_combo	**EON_Rank**
**BTP2 (YM 58483)**	ZINC000000591398	1.742	0.859	0.883	1	0.754	1.613	1
	ZINC000002999928	1.092	0.716	0.377	20	0.542	1.247	2
	ZINC000000004840	1.301	0.634	0.667	2	0.579	1.24	3
	ZINC000044607934	1.235	0.689	0.546	4	0.472	1.161	4
	ZINC000013813291	0.988	0.656	0.332	68	0.474	1.147	5
	ZINC000000016912	1.016	0.654	0.362	48	0.477	1.132	6
	ZINC000000598965	1.134	0.673	0.461	10	0.449	1.123	7
	ZINC000072232826	1.074	0.789	0.285	27	0.335	1.12	8
	ZINC000000061619	1.103	0.754	0.349	18	0.356	1.11	9
	ZINC000043206649	1.105	0.717	0.389	16	0.394	1.11	10
	ZINC000252672834	1.099	0.676	0.423	19	0.429	1.105	11
	ZINC000002320807	1.186	0.744	0.442	7	0.358	1.098	12
	ZINC000098052586	1.282	0.791	0.491	3	0.353	1.088	13
	ZINC000000005475	1.011	0.66	0.351	51	0.425	1.085	14
	ZINC000020509178	0.928	0.695	0.234	162	0.386	1.081	15
	ZINC000072317473	1.09	0.794	0.296	22	0.284	1.077	16
	ZINC000027101553	1.127	0.659	0.468	11	0.447	1.064	17
	ZINC000013815413	0.927	0.649	0.278	167	0.417	1.057	18
	ZINC000000600558	1.029	0.641	0.388	41	0.41	1.051	19
	ZINC000002570895	1.026	0.638	0.388	42	0.408	1.046	20
	ZINC000003809416	0.874	0.704	0.17	373	0.342	1.046	21
	ZINC000002391787	0.964	0.664	0.3	95	0.376	1.04	22
	ZINC000001542392	0.874	0.715	0.16	369	0.327	1.038	23
	ZINC000013512456	0.897	0.595	0.301	263	0.428	1.036	24
	ZINC000011852816	0.908	0.652	0.256	223	0.401	1.025	25
	ZINC000066099084	1.088	0.666	0.422	23	0.358	1.023	26
	ZINC000001386783	0.912	0.601	0.311	208	0.421	1.022	27
	ZINC000208398371	1.084	0.675	0.409	25	0.307	1.02	28
	ZINC000000881739	0.862	0.622	0.24	434	0.406	1.016	29
	ZINC000095616599	0.915	0.766	0.149	198	0.247	1.015	30
	ZINC000063298411	0.927	0.679	0.248	164	0.346	1.014	31
	ZINC000000007442	0.897	0.615	0.282	260	0.396	1.012	32
	ZINC000041739029	0.913	0.64	0.273	206	0.376	1.011	33
	ZINC000034884123	1.007	0.548	0.459	52	0.459	1.008	34
	ZINC000001843099	0.913	0.655	0.259	202	0.365	1.008	35

ZINC000066138253	0.949	0.678	0.27	126	0.313	0.991	36
ZINC000002757033	1.058	0.604	0.453	30	0.404	0.99	37
ZINC000095581007	0.961	0.651	0.311	101	0.339	0.989	38
ZINC000013862833	1.045	0.725	0.32	35	0.265	0.989	39
ZINC000000598952	1.056	0.673	0.383	31	0.313	0.986	40
ZINC000043203994	1.112	0.657	0.455	14	0.323	0.982	41
ZINC000000696906	0.978	0.65	0.328	75	0.314	0.975	42
ZINC000004521772	0.892	0.667	0.225	289	0.307	0.974	43
ZINC000066100415	0.873	0.674	0.199	379	0.3	0.974	44
ZINC000035816231	0.917	0.597	0.319	194	0.365	0.972	45
ZINC000001028145	1.193	0.723	0.47	6	0.245	0.97	46
ZINC000000116937	0.863	0.552	0.311	425	0.431	0.967	47
ZINC000040951471	0.862	0.613	0.249	433	0.353	0.966	48
ZINC000013479414	0.904	0.63	0.274	238	0.335	0.965	49
ZINC000059223284	0.87	0.62	0.251	386	0.343	0.962	50
ZINC000004358191	1.023	0.679	0.344	43	0.256	0.962	51
ZINC000013675378	0.858	0.737	0.12	463	0.218	0.956	52
ZINC000003818778	0.954	0.679	0.275	114	0.277	0.956	53
ZINC000003845641	0.859	0.638	0.221	455	0.306	0.951	54
ZINC000000096084	0.909	0.777	0.132	220	0.174	0.951	55
ZINC000003830986	0.963	0.72	0.243	96	0.23	0.951	56
ZINC000004365402	0.9	0.672	0.228	254	0.281	0.947	57
ZINC000002572463	0.863	0.761	0.101	426	0.186	0.947	58
ZINC000002244161	1.005	0.61	0.395	54	0.336	0.945	59
ZINC000000386312	0.88	0.61	0.271	348	0.337	0.943	60
ZINC000035794284	1.09	0.682	0.409	21	0.262	0.943	61
ZINC000252668237	1.016	0.64	0.377	47	0.302	0.942	62
ZINC000253476147	1.138	0.72	0.418	9	0.22	0.94	63
ZINC000018206988	0.944	0.71	0.233	134	0.227	0.937	64
ZINC000059102467	1.104	0.71	0.394	17	0.229	0.937	65
ZINC000095099975	0.869	0.656	0.213	393	0.305	0.937	66
ZINC000035323148	0.962	0.694	0.268	100	0.242	0.936	67
ZINC000000280804	0.904	0.705	0.199	239	0.236	0.933	68
ZINC000014982453	0.906	0.628	0.279	229	0.33	0.932	69
ZINC000000150552	1.047	0.479	0.568	34	0.45	0.929	70
ZINC000003941174	0.859	0.721	0.138	457	0.204	0.925	71
ZINC000000538658	0.969	0.568	0.4	86	0.324	0.925	72
ZINC000098052585	1.231	0.702	0.53	5	0.217	0.924	73
ZINC000000057237	0.871	0.639	0.232	383	0.286	0.924	74

ZINC000001753799	0.893	0.515	0.377	284	0.406	0.922	75
ZINC000072283454	0.904	0.666	0.237	237	0.312	0.922	76
ZINC000000538415	0.926	0.784	0.142	172	0.135	0.919	77
ZINC000003818808	0.88	0.646	0.233	349	0.27	0.916	78
ZINC000058649939	1.068	0.651	0.417	28	0.261	0.916	79
ZINC000008312780	0.954	0.497	0.457	115	0.428	0.916	80
ZINC000000538116	0.868	0.601	0.267	401	0.313	0.914	81
ZINC000012502280	0.9	0.591	0.309	247	0.318	0.909	82
ZINC000001530833	0.903	0.774	0.129	240	0.134	0.908	83
ZINC000038463473	0.893	0.692	0.2	283	0.254	0.907	84
ZINC000000008794	0.992	0.737	0.256	63	0.167	0.904	85
ZINC000038192558	0.898	0.593	0.306	257	0.299	0.903	86
ZINC000000003803	0.896	0.608	0.287	266	0.274	0.901	87
ZINC000072316228	0.864	0.706	0.158	420	0.193	0.9	88
ZINC000084669832	0.889	0.7	0.189	305	0.2	0.9	89
ZINC000002761895	0.993	0.732	0.261	62	0.165	0.897	90
ZINC000040875741	0.881	0.578	0.303	344	0.319	0.896	91
ZINC000003818628	0.855	0.544	0.311	477	0.351	0.895	92
ZINC000001553653	0.905	0.643	0.262	235	0.295	0.895	93
ZINC000000915717	0.957	0.691	0.265	108	0.202	0.893	94
ZINC000001024901	0.855	0.636	0.219	479	0.257	0.893	95
ZINC000001537834	0.867	0.703	0.164	404	0.196	0.893	96
ZINC000000601825	0.929	0.519	0.409	161	0.382	0.892	97
ZINC000000008815	0.865	0.651	0.214	414	0.269	0.883	98
ZINC000028951699	0.907	0.663	0.245	226	0.194	0.881	99
ZINC000004693575	0.918	0.697	0.221	190	0.186	0.879	100
ZINC000007992856	0.981	0.691	0.29	72	0.185	0.877	101
ZINC000001490477	0.973	0.573	0.4	80	0.262	0.877	102
ZINC000098052696	1.126	0.589	0.537	12	0.288	0.877	103
ZINC000001752601	0.857	0.526	0.331	467	0.342	0.872	104
ZINC000002719689	0.949	0.734	0.215	123	0.137	0.871	105
ZINC000003820709	0.888	0.636	0.251	310	0.234	0.871	106
ZINC000001533102	0.887	0.463	0.424	317	0.428	0.866	107
ZINC000000005994	0.949	0.539	0.41	124	0.327	0.866	108
ZINC000002522669	0.952	0.784	0.168	118	0.081	0.866	109
ZINC000000070468	0.864	0.599	0.265	419	0.266	0.864	110
ZINC000004247241	0.92	0.782	0.138	183	0.082	0.864	111
ZINC000004034906	0.957	0.711	0.246	107	0.152	0.863	112
ZINC000000140482	0.891	0.622	0.269	296	0.258	0.863	113

ZINC000021943479	1.006	0.664	0.343	53	0.198	0.862	114
ZINC000001851251	1.015	0.739	0.276	49	0.122	0.861	115
ZINC000002719825	0.921	0.719	0.202	179	0.141	0.86	116
ZINC000000001473	0.961	0.661	0.3	104	0.199	0.86	117
ZINC000043207558	0.92	0.635	0.285	182	0.224	0.86	118
ZINC000004251680	0.946	0.792	0.154	131	0.065	0.857	119
ZINC000003806413	0.919	0.647	0.272	186	0.212	0.855	120
ZINC000022454188	0.909	0.769	0.141	219	0.086	0.855	121
ZINC000008648798	0.972	0.683	0.289	84	0.148	0.855	122
ZINC000001481850	0.923	0.753	0.17	176	0.096	0.854	123
ZINC000003787086	1.085	0.799	0.286	24	0.048	0.854	124
ZINC000009458454	0.884	0.528	0.356	329	0.33	0.854	125
ZINC000004693574	0.912	0.689	0.223	209	0.168	0.853	126
ZINC000147625631	0.865	0.603	0.263	413	0.235	0.852	127
ZINC000254286814	0.875	0.716	0.159	367	0.117	0.852	128
ZINC000013527116	0.909	0.691	0.218	218	0.158	0.849	129
ZINC000003872076	0.917	0.669	0.248	191	0.179	0.847	130
ZINC000000605984	0.886	0.69	0.195	322	0.156	0.847	131
ZINC000002507598	0.878	0.73	0.148	355	0.117	0.847	132
ZINC000000567816	0.965	0.575	0.391	93	0.297	0.846	133
ZINC000100067972	0.867	0.6	0.267	406	0.286	0.846	134
ZINC000013612035	1.149	0.679	0.47	8	0.166	0.845	135
ZINC000000608235	0.86	0.619	0.241	452	0.233	0.843	136
ZINC000005114736	0.887	0.764	0.123	318	0.078	0.842	137
ZINC000027090735	1.078	0.738	0.34	26	0.104	0.842	138
ZINC000087493318	0.968	0.545	0.423	88	0.291	0.842	139
ZINC000140247580	1.001	0.626	0.375	56	0.215	0.841	140
ZINC000003812944	0.955	0.562	0.394	111	0.278	0.839	141
ZINC000001040357	0.906	0.702	0.204	233	0.111	0.839	142
ZINC000022028887	0.926	0.783	0.143	170	0.056	0.839	143
ZINC000031424814	0.95	0.68	0.27	121	0.156	0.838	144
ZINC000003818815	0.948	0.745	0.203	130	0.083	0.838	145
ZINC000000004041	0.989	0.712	0.277	66	0.122	0.837	146
ZINC000002575699	0.977	0.535	0.442	76	0.342	0.836	147
ZINC000002141876	0.883	0.692	0.19	337	0.142	0.834	148
ZINC000006273129	0.908	0.76	0.149	221	0.073	0.833	149
ZINC000003951541	0.911	0.664	0.247	215	0.168	0.832	150
ZINC000100001820	0.857	0.699	0.158	472	0.132	0.831	151
ZINC000010773901	0.931	0.495	0.435	155	0.303	0.831	152

ZINC000252675735	0.853	0.569	0.284	495	0.269	0.83	153
ZINC000095619105	0.939	0.716	0.224	141	0.114	0.83	154
ZINC000013831242	0.941	0.682	0.259	138	0.15	0.829	155
ZINC000001248847	0.895	0.741	0.154	270	0.086	0.828	156
ZINC000022442521	0.901	0.761	0.14	243	0.066	0.827	157
ZINC000007992851	0.867	0.523	0.344	407	0.292	0.827	158
ZINC000000110861	0.873	0.554	0.319	377	0.265	0.826	159
ZINC000003995874	0.967	0.656	0.311	92	0.222	0.826	160
ZINC000026665488	0.852	0.692	0.16	497	0.131	0.825	161
ZINC000018190814	0.873	0.693	0.18	378	0.132	0.823	162
ZINC000094566092	0.892	0.648	0.244	288	0.174	0.822	163
ZINC000000006300	0.853	0.596	0.257	492	0.224	0.82	164
ZINC000003584715	0.99	0.708	0.282	65	0.112	0.82	165
ZINC000039258365	0.878	0.645	0.233	360	0.172	0.817	166
ZINC000001488270	0.9	0.639	0.261	249	0.16	0.816	167
ZINC000220951188	1	0.783	0.217	58	0.032	0.816	168
ZINC000000643233	0.882	0.647	0.235	338	0.162	0.816	169
ZINC000003995738	0.917	0.589	0.328	193	0.225	0.814	170
ZINC000029938125	0.9	0.744	0.156	248	0.07	0.814	171
ZINC000013815318	0.917	0.727	0.19	192	0.094	0.813	172
ZINC000001248848	0.883	0.729	0.153	333	0.083	0.813	173
ZINC000013984603	0.98	0.618	0.362	74	0.194	0.812	174
ZINC000098052553	0.906	0.663	0.243	231	0.149	0.812	175
ZINC000025758996	0.87	0.643	0.227	387	0.116	0.812	176
ZINC000000630739	0.93	0.616	0.314	157	0.196	0.812	177
ZINC000067980453	1.04	0.671	0.369	37	0.139	0.81	178
ZINC000004543674	0.863	0.616	0.248	422	0.193	0.809	179
ZINC000022454193	0.891	0.751	0.14	292	0.058	0.809	180
ZINC000000577115	0.938	0.711	0.227	143	0.097	0.808	181
ZINC000035999346	0.967	0.685	0.283	90	0.125	0.807	182
ZINC000000001672	0.868	0.644	0.223	403	0.162	0.807	183
ZINC000004034905	0.961	0.709	0.252	102	0.097	0.806	184
ZINC000000004448	0.863	0.606	0.258	424	0.165	0.805	185
ZINC000000599734	0.895	0.764	0.132	269	0.041	0.804	186
ZINC000095593440	0.886	0.616	0.27	321	0.188	0.804	187
ZINC000021288919	0.919	0.616	0.303	185	0.187	0.804	188
ZINC000068250805	0.918	0.652	0.266	188	0.151	0.804	189
ZINC000097306095	1.017	0.782	0.235	46	0.021	0.803	190
ZINC000000968328	0.88	0.71	0.171	346	0.092	0.801	191

ZINC000000014457	0.913	0.749	0.164	204	0.051	0.8	192
ZINC000019364229	0.911	0.751	0.161	214	0.049	0.8	193
ZINC000065731313	0.883	0.658	0.226	332	0.132	0.799	194
ZINC000147419870	0.878	0.628	0.25	357	0.17	0.799	195
ZINC000064696513	0.919	0.68	0.24	187	0.119	0.799	196
ZINC000008648797	0.948	0.683	0.265	129	0.115	0.797	197
ZINC000043122151	0.866	0.468	0.399	410	0.267	0.797	198
ZINC000003960083	0.983	0.605	0.378	70	0.178	0.797	199
ZINC000053151228	0.972	0.669	0.303	83	0.127	0.796	200
ZINC000003777909	0.86	0.688	0.172	449	0.108	0.796	201
ZINC000019364230	0.918	0.752	0.166	189	0.044	0.796	202
ZINC000001843091	0.953	0.696	0.257	117	0.099	0.795	203
ZINC000053122805	0.883	0.738	0.144	335	0.049	0.794	204
ZINC000000000706	1.018	0.688	0.33	45	0.105	0.793	205
ZINC000053064442	0.911	0.663	0.248	216	0.12	0.793	206
ZINC000252286763	0.972	0.571	0.401	81	0.219	0.791	207
ZINC000003874496	1.039	0.594	0.445	39	0.174	0.788	208
ZINC000000724635	0.962	0.53	0.432	99	0.264	0.788	209
ZINC000096024631	0.875	0.659	0.215	368	0.127	0.787	210
ZINC000001238258	0.859	0.691	0.168	456	0.096	0.786	211
ZINC000008614303	0.891	0.568	0.323	299	0.218	0.786	212
ZINC000000602170	0.927	0.666	0.261	166	0.119	0.786	213
ZINC000052957106	0.931	0.654	0.276	154	0.13	0.784	214
ZINC000000579013	0.895	0.642	0.253	276	0.14	0.782	215
ZINC000066251730	0.893	0.706	0.187	282	0.075	0.781	216
ZINC000002011294	0.892	0.708	0.185	287	0.07	0.778	217
ZINC000000538354	0.876	0.735	0.141	363	0.041	0.778	218
ZINC000035793305	0.89	0.684	0.206	301	0.092	0.776	219
ZINC000035636062	0.937	0.644	0.293	148	0.13	0.774	220
ZINC000095616600	0.898	0.731	0.166	259	0.055	0.773	221
ZINC000100086534	0.87	0.611	0.259	389	0.162	0.772	222
ZINC000013981546	0.868	0.577	0.291	400	0.194	0.771	223
ZINC000022309498	1.106	0.726	0.38	15	0.044	0.771	224
ZINC000137706092	0.895	0.749	0.146	274	0.021	0.771	225
ZINC000000620751	0.913	0.688	0.225	205	0.082	0.77	226
ZINC000005020529	0.916	0.726	0.19	197	0.044	0.77	227
ZINC000003810035	0.971	0.7	0.271	85	0.059	0.77	228
ZINC000195355080	0.868	0.669	0.199	399	0.101	0.77	229
ZINC000000057848	0.895	0.615	0.279	277	0.163	0.77	230

ZINC000084740552	0.974	0.673	0.301	79	0.104	0.769	231
ZINC000000598852	0.862	0.686	0.175	435	0.076	0.769	232
ZINC000207660265	0.968	0.595	0.373	89	0.173	0.769	233
ZINC000027647189	0.983	0.686	0.297	69	0.083	0.769	234
ZINC000003616630	0.883	0.648	0.235	334	0.12	0.768	235
ZINC000072190123	0.889	0.736	0.153	304	0.032	0.768	236
ZINC000004632523	0.961	0.705	0.256	105	0.062	0.767	237
ZINC000019632628	0.969	0.733	0.235	87	0.033	0.767	238
ZINC000072317687	0.896	0.679	0.216	268	0.087	0.766	239
ZINC000018115268	0.93	0.584	0.346	156	0.181	0.765	240
ZINC000007485953	0.915	0.665	0.25	199	0.121	0.765	241
ZINC000000601841	0.896	0.645	0.251	265	0.107	0.765	242
ZINC000026396245	0.923	0.644	0.279	177	0.13	0.764	243
ZINC000001532364	0.894	0.616	0.278	279	0.138	0.763	244
ZINC000001893129	0.852	0.701	0.151	498	0.063	0.763	245
ZINC000003784077	0.863	0.634	0.229	427	0.129	0.763	246
ZINC000002803051	0.901	0.608	0.293	245	0.155	0.763	247
ZINC000002483738	0.956	0.687	0.269	109	0.075	0.763	248
ZINC000000014551	0.908	0.663	0.245	225	0.076	0.762	249
ZINC000001532333	0.884	0.619	0.265	327	0.144	0.762	250
ZINC000001540772	0.912	0.67	0.242	213	0.089	0.761	251
ZINC000095554357	0.953	0.608	0.345	116	0.152	0.761	252
ZINC000143574007	0.886	0.689	0.196	323	0.041	0.76	253
ZINC000002169424	0.867	0.705	0.162	405	0.055	0.76	254
ZINC000026174383	0.865	0.661	0.204	416	0.051	0.76	255
ZINC000078938112	0.922	0.621	0.3	178	0.139	0.76	256
ZINC000003812136	0.858	0.687	0.171	464	0.073	0.76	257
ZINC000000598040	0.854	0.635	0.219	483	0.125	0.759	258
ZINC000004632541	0.942	0.672	0.27	137	0.086	0.758	259
ZINC000059103853	0.949	0.747	0.202	125	0.035	0.758	260
ZINC000095616601	0.901	0.654	0.248	242	0.103	0.757	261
ZINC000003954923	0.9	0.598	0.302	250	0.157	0.756	262
ZINC000001550170	0.906	0.608	0.299	230	0.175	0.755	263
ZINC000003812549	0.955	0.697	0.258	112	0.058	0.755	264
ZINC000033980255	0.958	0.613	0.345	106	0.141	0.755	265
ZINC000001846593	0.86	0.62	0.24	451	0.126	0.755	266
ZINC000052968892	0.943	0.63	0.313	135	0.124	0.754	267
ZINC000000005423	0.961	0.699	0.262	103	0.055	0.754	268
ZINC000000591392	0.857	0.717	0.14	471	0.032	0.752	269

ZINC000001997139	0.864	0.534	0.33	418	0.26	0.751	270
ZINC000088131135	0.887	0.673	0.214	313	0.075	0.751	271
ZINC000000538312	1.065	0.634	0.431	29	0.116	0.75	272
ZINC000000007547	0.861	0.671	0.19	438	0.079	0.75	273
ZINC000095642812	0.962	0.622	0.341	98	0.128	0.749	274
ZINC000002008866	0.932	0.575	0.357	153	0.166	0.749	275
ZINC000252585174	0.879	0.618	0.262	352	0.131	0.748	276
ZINC000100080852	0.895	0.563	0.333	271	0.16	0.748	277
ZINC000000191638	0.967	0.68	0.287	91	0.071	0.747	278
ZINC000000191636	0.965	0.678	0.287	94	0.068	0.746	279
ZINC000226098129	0.862	0.548	0.314	429	0.186	0.746	280
ZINC000003683815	1.121	0.682	0.439	13	0.064	0.746	281
ZINC000001530571	0.863	0.697	0.167	423	0.049	0.745	282
ZINC000001820907	0.914	0.655	0.258	200	0.09	0.745	283
ZINC000002011295	0.881	0.702	0.179	343	0.042	0.744	284
ZINC000098210306	0.862	0.653	0.209	428	0.081	0.744	285
ZINC000000538069	0.942	0.554	0.389	136	0.127	0.744	286
ZINC000003872044	0.896	0.639	0.257	264	0.094	0.744	287
ZINC000004214700	0.993	0.601	0.392	61	0.142	0.743	288
ZINC000081213892	0.874	0.694	0.18	372	0.049	0.743	289
ZINC000003993799	0.992	0.634	0.358	64	0.114	0.742	290
ZINC000003778381	0.935	0.725	0.209	151	0.017	0.742	291
ZINC000003995739	0.895	0.565	0.33	275	0.177	0.742	292
ZINC000086021943	0.912	0.725	0.187	212	0.016	0.741	293
ZINC000000865051	0.929	0.749	0.181	158	0.079	0.741	294
ZINC000002030426	0.883	0.68	0.202	336	0.064	0.74	295
ZINC000002563946	0.884	0.648	0.236	331	0.093	0.74	296
ZINC000252668985	0.945	0.654	0.291	132	0.077	0.74	297
ZINC000043152493	0.995	0.58	0.415	59	0.214	0.74	298
ZINC000038214379	0.897	0.583	0.314	261	0.156	0.739	299
ZINC000095921314	0.87	0.706	0.164	391	0.032	0.738	300
ZINC000001999423	0.935	0.7	0.235	150	0.037	0.738	301
ZINC000003938686	0.857	0.739	0.117	473	-0.011	0.737	302
ZINC000003834020	0.884	0.73	0.154	326	0.006	0.737	303
ZINC000004214955	0.866	0.706	0.161	408	0.03	0.736	304
ZINC000006804365	0.862	0.619	0.243	431	0.117	0.736	305
ZINC000003929366	0.938	0.608	0.33	144	0.127	0.735	306
ZINC000036382858	0.949	0.724	0.224	127	0.014	0.735	307
ZINC000001549377	1.052	0.673	0.379	32	0.062	0.735	308

ZINC000000601958	0.891	0.663	0.229	293	0.071	0.734	309
ZINC000100002044	0.937	0.663	0.274	149	0.07	0.733	310
ZINC000003860715	0.902	0.71	0.191	241	0.015	0.732	311
ZINC000003872275	0.874	0.598	0.276	371	0.136	0.732	312
ZINC000003995603	0.861	0.57	0.291	444	0.162	0.732	313
ZINC000028710494	0.897	0.621	0.277	262	0.111	0.732	314
ZINC000001540228	0.895	0.535	0.36	273	0.239	0.731	315
ZINC000033359230	0.939	0.674	0.265	140	0.057	0.731	316
ZINC000095803215	1.03	0.692	0.338	40	0.039	0.73	317
ZINC000049582255	0.924	0.649	0.274	175	0.081	0.73	318
ZINC000002557963	0.854	0.618	0.236	487	0.111	0.73	319
ZINC000043277624	0.871	0.739	0.131	385	-0.007	0.729	320
ZINC000100015335	0.88	0.704	0.176	351	0.033	0.729	321
ZINC000000602847	0.938	0.71	0.228	146	0.019	0.729	322
ZINC000000537931	0.87	0.615	0.255	388	0.114	0.729	323
ZINC000003811810	0.855	0.708	0.146	480	0.001	0.728	324
ZINC000038332063	0.9	0.683	0.217	253	0.045	0.727	325
ZINC000002903149	0.854	0.605	0.248	488	0.155	0.727	326
ZINC000086003193	0.907	0.711	0.196	227	0.019	0.727	327
ZINC000013813297	0.913	0.65	0.264	201	0.077	0.726	328
ZINC000019606670	0.913	0.519	0.394	203	0.207	0.726	329
ZINC000071773472	0.919	0.721	0.199	184	0.005	0.726	330
ZINC000071331761	0.916	0.699	0.217	196	0.026	0.725	331
ZINC000252668986	0.937	0.646	0.291	147	0.076	0.725	332
ZINC000003810797	0.893	0.654	0.24	281	0.069	0.725	333
ZINC000215797983	0.944	0.648	0.296	133	0.073	0.725	334
ZINC000098052501	0.938	0.644	0.295	142	0.077	0.724	335
ZINC000000038235	0.927	0.605	0.322	165	0.118	0.724	336
ZINC000001820906	0.894	0.636	0.258	278	0.087	0.724	337
ZINC000143737811	1.004	0.816	0.188	55	-0.093	0.723	338
ZINC000000599925	1.051	0.643	0.408	33	0.081	0.723	339
ZINC000000538550	0.891	0.611	0.28	297	0.112	0.723	340
ZINC000018203737	0.882	0.55	0.332	340	0.172	0.722	341
ZINC000003964325	0.858	0.655	0.203	461	0.067	0.722	342
ZINC000001642602	1.021	0.61	0.411	44	0.111	0.722	343
ZINC000000538314	0.859	0.584	0.275	459	0.134	0.721	344
ZINC000036382102	0.853	0.674	0.179	494	0.043	0.72	345
ZINC000000537914	0.887	0.78	0.107	315	-0.048	0.719	346
ZINC000003794575	0.881	0.677	0.204	342	0.035	0.718	347

ZINC000003990436	0.868	0.638	0.229	402	0.08	0.718	348
ZINC000007992852	0.885	0.687	0.198	325	0.022	0.717	349
ZINC000003820049	0.912	0.698	0.214	211	0.04	0.717	350
ZINC000038143010	0.949	0.725	0.224	122	0.009	0.716	351
ZINC000026817456	0.857	0.657	0.2	468	0.058	0.716	352
ZINC000013819807	0.854	0.627	0.227	484	0.088	0.715	353
ZINC000043132689	0.879	0.692	0.188	353	0.022	0.714	354
ZINC000028228318	0.875	0.668	0.208	365	0.048	0.713	355
ZINC000003923836	0.855	0.621	0.234	481	0.085	0.712	356
ZINC000001532365	0.861	0.583	0.278	442	0.12	0.712	357
ZINC000068207669	0.878	0.593	0.285	358	0.119	0.711	358
ZINC000001512677	0.9	0.706	0.194	251	0.004	0.711	359
ZINC000029043262	0.856	0.612	0.244	475	0.098	0.711	360
ZINC000028863086	0.926	0.649	0.278	171	0.085	0.71	361
ZINC000003941698	0.899	0.725	0.174	256	-0.016	0.709	362
ZINC000002009671	0.951	0.607	0.344	119	0.101	0.709	363
ZINC000095644391	0.857	0.718	0.139	470	-0.002	0.708	364
ZINC000000275584	0.865	0.665	0.2	415	0.042	0.707	365
ZINC000000755692	0.972	0.769	0.203	82	-0.063	0.706	366
ZINC000098210305	0.86	0.497	0.363	448	0.209	0.706	367
ZINC000068251494	0.977	0.593	0.385	77	0.113	0.706	368
ZINC000023640382	0.878	0.641	0.236	359	0.063	0.705	369
ZINC000025756875	0.879	0.597	0.282	354	0.086	0.705	370
ZINC000037866151	0.908	0.546	0.362	222	0.21	0.704	371
ZINC000009212098	0.886	0.503	0.383	320	0.202	0.703	372
ZINC000003816409	0.896	0.631	0.265	267	0.071	0.702	373
ZINC000005167145	0.908	0.677	0.231	224	0.025	0.702	374
ZINC000068267183	0.852	0.513	0.338	500	0.199	0.702	375
ZINC000013676140	0.888	0.699	0.189	307	0.002	0.701	376
ZINC000044559762	1.012	0.716	0.295	50	-0.015	0.701	377
ZINC000000257456	0.888	0.713	0.175	306	-0.013	0.7	378
ZINC000019683150	0.926	0.634	0.293	168	0.065	0.699	379
ZINC000003995998	0.884	0.686	0.198	328	0.013	0.699	380
ZINC000019774244	0.864	0.608	0.257	417	0.084	0.698	381
ZINC000026153621	0.894	0.703	0.191	280	-0.006	0.698	382
ZINC000038463474	0.95	0.646	0.304	120	0.051	0.697	383
ZINC000003827164	0.907	0.618	0.289	228	0.078	0.696	384
ZINC000040163635	0.926	0.673	0.253	169	0.022	0.696	385
ZINC000043132686	0.88	0.677	0.203	350	0.019	0.696	386

ZINC000003936850	0.901	0.688	0.213	244	-0.004	0.695	387
ZINC000000359757	0.891	0.631	0.26	295	0.063	0.695	388
ZINC000003995996	0.901	0.702	0.199	246	-0.01	0.693	389
ZINC000043132688	0.873	0.709	0.164	375	-0.017	0.692	390
ZINC000013829356	0.853	0.641	0.212	493	0.1	0.691	391
ZINC000000006097	0.905	0.63	0.275	234	0.059	0.69	392
ZINC000003871759	0.86	0.754	0.106	454	-0.064	0.689	393
ZINC000095560373	0.869	0.628	0.241	394	0.053	0.689	394
ZINC000000599006	0.873	0.547	0.327	374	0.131	0.687	395
ZINC000001534779	0.888	0.614	0.274	312	0.072	0.687	396
ZINC000004954743	0.877	0.627	0.25	362	0.059	0.686	397
ZINC000060119908	1.001	0.666	0.335	57	0.019	0.685	398
ZINC000003812548	0.873	0.585	0.287	380	0.107	0.685	399
ZINC000000257471	0.872	0.698	0.174	381	-0.015	0.683	400
ZINC000000216435	0.861	0.589	0.273	439	0.093	0.682	401
ZINC000071773588	0.87	0.537	0.332	390	0.151	0.682	402
ZINC000052541473	0.861	0.685	0.175	445	-0.004	0.682	403
ZINC000004096811	0.861	0.641	0.22	441	0.04	0.682	404
ZINC000028332661	0.895	0.613	0.282	272	0.068	0.681	405
ZINC000013829437	0.869	0.706	0.162	396	-0.036	0.681	406
ZINC000001489479	0.869	0.538	0.33	398	0.155	0.68	407
ZINC000096271026	0.887	0.646	0.241	314	0.033	0.679	408
ZINC000095567261	0.89	0.692	0.198	300	-0.019	0.676	409
ZINC000011687445	0.916	0.723	0.193	195	-0.047	0.676	410
ZINC000003978987	0.861	0.624	0.238	440	0.052	0.676	411
ZINC000000602172	0.983	0.612	0.371	71	0.07	0.676	412
ZINC000004533909	0.866	0.668	0.198	411	0.006	0.674	413
ZINC000034042470	1.043	0.7	0.344	36	0.015	0.674	414
ZINC000001530776	0.861	0.606	0.255	443	0.066	0.674	415
ZINC000014959960	0.878	0.675	0.203	356	-0.002	0.673	416
ZINC000004070868	0.865	0.751	0.115	412	-0.078	0.673	417
ZINC000003987355	0.925	0.552	0.373	173	0.121	0.673	418
ZINC000003995923	0.938	0.584	0.354	145	0.089	0.673	419
ZINC000095099976	0.898	0.685	0.213	258	0.039	0.673	420
ZINC000043201623	0.974	0.631	0.343	78	0.041	0.672	421
ZINC000043205680	0.862	0.612	0.25	432	0.029	0.671	422
ZINC000002383344	0.928	0.527	0.401	163	0.144	0.671	423
ZINC000019537374	0.981	0.652	0.329	73	0.019	0.671	424
ZINC000025427051	0.912	0.676	0.237	210	-0.006	0.67	425

ZINC000052607342	0.876	0.649	0.227	364	0.02	0.669	426
ZINC000000603862	1.04	0.682	0.357	38	0.027	0.669	427
ZINC000001536786	0.875	0.645	0.23	366	0.024	0.669	428
ZINC000000598193	0.858	0.654	0.204	465	0.014	0.668	429
ZINC000001488294	0.852	0.522	0.33	496	0.14	0.666	430
ZINC000000597570	0.892	0.57	0.321	291	0.096	0.666	431
ZINC000001481956	0.9	0.52	0.38	252	0.146	0.666	432
ZINC000040861329	0.871	0.582	0.289	384	0.083	0.665	433
ZINC000001489460	0.874	0.639	0.235	370	0.025	0.663	434
ZINC000013130939	0.885	0.676	0.209	324	-0.014	0.663	435
ZINC000005021635	0.866	0.594	0.273	409	0.066	0.66	436
ZINC000003876186	0.921	0.698	0.223	181	-0.04	0.658	437
ZINC000018193448	0.882	0.605	0.278	339	0.039	0.657	438
ZINC000005956176	0.854	0.663	0.191	486	-0.008	0.656	439
ZINC000002557962	0.86	0.576	0.284	447	0.078	0.654	440
ZINC000026381513	0.877	0.586	0.291	361	0.073	0.653	441
ZINC000000601274	0.862	0.583	0.279	437	0.065	0.653	442
ZINC000014954393	0.906	0.69	0.215	232	-0.039	0.651	443
ZINC000003881648	0.852	0.638	0.214	499	0.011	0.649	444
ZINC000000527386	0.89	0.668	0.222	302	-0.02	0.648	445
ZINC000003792990	0.869	0.501	0.368	395	0.146	0.648	446
ZINC000040414458	0.913	0.647	0.265	207	-0.006	0.644	447
ZINC000033246138	0.873	0.623	0.25	376	0.017	0.642	448
ZINC000000013635	0.91	0.551	0.358	217	0.087	0.639	449
ZINC000003959835	0.921	0.572	0.349	180	0.067	0.639	450
ZINC000000853334	0.956	0.762	0.194	110	-0.125	0.636	451
ZINC000043207268	0.858	0.596	0.262	462	0.04	0.636	452
ZINC000013813280	0.864	0.674	0.189	421	-0.04	0.635	453
ZINC000000003381	0.88	0.459	0.421	347	0.173	0.632	454
ZINC000001854671	0.939	0.719	0.22	139	-0.088	0.632	455
ZINC000003801104	0.887	0.639	0.248	316	-0.008	0.631	456
ZINC000026153628	0.853	0.646	0.207	491	-0.017	0.63	457
ZINC000040414461	0.948	0.573	0.375	128	0.056	0.629	458
ZINC000000596951	0.891	0.598	0.293	294	0.028	0.627	459
ZINC000000626716	0.859	0.688	0.171	458	-0.062	0.626	460
ZINC000051133897	0.892	0.634	0.258	290	-0.01	0.624	461
ZINC000000943071	0.962	0.601	0.362	97	-0.004	0.623	462
ZINC000007992835	0.857	0.602	0.256	466	0.025	0.622	463
ZINC000095583412	0.861	0.675	0.186	446	-0.052	0.621	464

ZINC000040979996	0.899	0.726	0.174	255	-0.07	0.621	465
ZINC000008687060	0.888	0.699	0.189	308	-0.08	0.619	466
ZINC000000602675	0.855	0.748	0.107	478	-0.142	0.616	467
ZINC000072315409	0.929	0.677	0.252	160	-0.037	0.616	468
ZINC000000000797	0.893	0.724	0.168	285	-0.11	0.615	469
ZINC000003871842	0.889	0.582	0.308	303	0.029	0.61	470
ZINC000000597739	0.884	0.514	0.369	330	0.093	0.608	471
ZINC000000898145	0.86	0.635	0.225	450	-0.029	0.606	472
ZINC000094566093	0.904	0.674	0.23	236	-0.07	0.604	473
ZINC000013829336	0.855	0.602	0.253	476	-0.004	0.604	474
ZINC000245240199	0.954	0.521	0.434	113	0.081	0.602	475
ZINC000034660430	0.862	0.572	0.29	430	0.03	0.601	476
ZINC000002119975	0.88	0.646	0.234	345	-0.023	0.595	477
ZINC000000851778	0.854	0.643	0.212	482	-0.048	0.595	478
ZINC000000092904	0.858	0.668	0.191	460	-0.078	0.59	479
ZINC000013108134	0.995	0.615	0.379	60	-0.028	0.587	480
ZINC000008397532	0.872	0.594	0.278	382	-0.009	0.585	481
ZINC000252675978	0.888	0.552	0.335	309	0.031	0.584	482
ZINC000000206382	0.891	0.638	0.253	298	-0.059	0.579	483
ZINC000026961992	0.857	0.641	0.216	469	-0.063	0.579	484
ZINC000001751810	0.888	0.62	0.268	311	-0.038	0.574	485
ZINC000247654071	0.853	0.463	0.39	490	0.111	0.574	486
ZINC000098208332	0.929	0.611	0.318	159	-0.039	0.572	487
ZINC000027646423	0.854	0.607	0.247	485	-0.041	0.567	488
ZINC000005853925	0.854	0.597	0.257	489	-0.032	0.565	489
ZINC000100036051	0.886	0.613	0.274	319	-0.051	0.562	490
ZINC000001481754	0.924	0.61	0.314	174	-0.062	0.548	491
ZINC000006631357	0.935	0.656	0.279	152	-0.115	0.537	492
ZINC000013473523	0.869	0.617	0.252	397	-0.084	0.533	493
ZINC000098052552	0.87	0.677	0.192	392	-0.154	0.523	494
ZINC000033982008	0.893	0.56	0.332	286	-0.045	0.515	495
ZINC000098052551	0.856	0.667	0.189	474	-0.174	0.506	496
ZINC000040977335	0.882	0.604	0.277	341	-0.082	0.496	497
ZINC000098052567	0.86	0.533	0.326	453	-0.069	0.479	498
ZINC000001541801	0.989	0.572	0.417	67	-0.104	0.468	499
ZINC000000035804	0.862	0.586	0.276	436	-0.129	0.457	500

Supplementary Table 2

ROC and EON based ranking of hits from Pyr6-basd screening of MedChemExpress library

		ROC related				EON related		
	zinc_id	TanimotoCombo	ShapeTanimoto	ColorTanimoto	**ROC Rank**	EON_ET_pb	EON_ET_combo	**EON_Rank**
**Pyr6**	ZINC000002022634	1.677	0.805	0.872	1	0.706	1.511	1
	ZINC000002999928	1.123	0.714	0.409	8	0.724	1.438	2
	ZINC000035794284	1.111	0.67	0.442	9	0.533	1.195	3
	ZINC000011678567	1.048	0.7	0.348	20	0.486	1.186	4
	ZINC000020509178	0.975	0.729	0.246	61	0.447	1.176	5
	ZINC000036690025	0.857	0.623	0.234	390	0.506	1.163	6
	ZINC000167006010	1.069	0.678	0.39	15	0.477	1.155	7
	ZINC000000592419	1.146	0.645	0.501	7	0.519	1.148	8
	ZINC000095578650	0.932	0.613	0.319	138	0.518	1.131	9
	ZINC000002583789	0.873	0.541	0.332	327	0.551	1.124	10
	ZINC000000004840	1.265	0.637	0.628	3	0.457	1.123	11
	ZINC000000155234	1.035	0.59	0.444	22	0.528	1.119	12
	ZINC000004212651	0.997	0.691	0.306	47	0.423	1.114	13
	ZINC000038141430	0.919	0.652	0.267	174	0.453	1.105	14
	ZINC000013603907	0.922	0.669	0.253	168	0.431	1.105	15
	ZINC000002483738	1.002	0.715	0.286	45	0.388	1.103	16
	ZINC000058649939	1.089	0.642	0.447	12	0.431	1.101	17
	ZINC000003121158	0.959	0.679	0.28	82	0.42	1.099	18
	ZINC000004676424	0.842	0.636	0.206	475	0.459	1.095	19
	ZINC000095556606	0.991	0.672	0.319	51	0.397	1.071	20
	ZINC000014982453	0.946	0.645	0.302	111	0.424	1.069	21
	ZINC000058649826	1.017	0.627	0.39	33	0.484	1.065	22
	ZINC000002541692	0.853	0.674	0.179	411	0.394	1.065	23
	ZINC000000538658	1.051	0.614	0.437	18	0.412	1.061	24
	ZINC000253387881	0.954	0.678	0.276	94	0.377	1.055	25
	ZINC000002565087	0.925	0.551	0.374	156	0.502	1.053	26
	ZINC000072317473	0.974	0.717	0.257	62	0.333	1.05	27
	ZINC000043206649	1.082	0.668	0.414	13	0.378	1.047	28
	ZINC000013986943	0.918	0.589	0.329	177	0.461	1.045	29
	ZINC000009576498	0.878	0.693	0.185	302	0.352	1.045	30
	ZINC000095616600	0.918	0.802	0.116	176	0.243	1.044	31
	ZINC000002244161	1.013	0.586	0.426	35	0.463	1.042	32
	ZINC000000567816	1.018	0.582	0.436	31	0.451	1.04	33
	ZINC000002047743	0.913	0.724	0.19	184	0.311	1.035	34
	ZINC000006718813	0.947	0.666	0.281	110	0.38	1.034	35

ZINC000000001473	1.024	0.684	0.34	30	0.348	1.031	36
ZINC000199564252	1.33	0.854	0.477	2	0.172	1.026	37
ZINC000000593415	1.011	0.678	0.332	36	0.377	1.025	38
ZINC000005137268	0.867	0.68	0.187	351	0.345	1.025	39
ZINC000000477459	0.851	0.601	0.25	419	0.421	1.022	40
ZINC000011677837	0.884	0.756	0.128	281	0.265	1.021	41
ZINC000068247898	1.033	0.654	0.379	25	0.363	1.017	42
ZINC000013512456	0.973	0.64	0.333	64	0.429	1.013	43
ZINC000028951699	0.925	0.668	0.256	160	0.344	1.012	44
ZINC000003976469	0.88	0.584	0.296	298	0.428	1.012	45
ZINC000001489430	0.864	0.611	0.252	365	0.396	1.008	46
ZINC000169347551	0.912	0.749	0.163	188	0.261	1.007	47
ZINC000003938688	0.84	0.678	0.162	484	0.294	1.005	48
ZINC000004358191	1.099	0.723	0.376	10	0.28	1.003	49
ZINC000004365402	0.899	0.652	0.247	217	0.354	0.997	50
ZINC000013985502	1.093	0.672	0.421	11	0.325	0.997	51
ZINC000000150552	1.045	0.422	0.623	21	0.534	0.996	52
ZINC000000000602	0.892	0.484	0.407	248	0.511	0.996	53
ZINC000005924265	0.977	0.552	0.425	59	0.425	0.996	54
ZINC000001494180	0.907	0.582	0.325	193	0.391	0.991	55
ZINC000012502280	0.916	0.584	0.332	179	0.404	0.989	56
ZINC000034642570	0.897	0.689	0.207	228	0.312	0.984	57
ZINC000004428527	1.003	0.651	0.352	44	0.34	0.983	58
ZINC000098052696	1.235	0.613	0.622	6	0.368	0.981	59
ZINC000065731313	0.944	0.689	0.254	117	0.297	0.98	60
ZINC000117645501	0.905	0.681	0.224	199	0.325	0.98	61
ZINC000003875348	0.884	0.612	0.272	278	0.377	0.979	62
ZINC000000600292	0.862	0.557	0.305	371	0.423	0.978	63
ZINC000003977990	0.904	0.649	0.256	202	0.329	0.978	64
ZINC000002526787	0.914	0.656	0.258	182	0.322	0.977	65
ZINC000003806413	0.956	0.662	0.294	88	0.321	0.977	66
ZINC000001549363	0.87	0.674	0.197	335	0.289	0.974	67
ZINC000043206812	0.847	0.565	0.282	440	0.411	0.973	68
ZINC000095593440	0.915	0.537	0.377	180	0.434	0.972	69
ZINC000003818778	0.898	0.679	0.219	222	0.323	0.97	70
ZINC000000725713	0.93	0.578	0.352	145	0.387	0.965	71
ZINC000009458454	0.888	0.515	0.373	259	0.426	0.964	72
ZINC000000140471	0.964	0.624	0.339	76	0.323	0.963	73
ZINC000035996961	1.003	0.712	0.292	42	0.248	0.96	74

ZINC000000244029	1.006	0.611	0.396	41	0.349	0.96	75
ZINC000000116937	0.887	0.555	0.332	260	0.403	0.959	76
ZINC000000602170	0.956	0.587	0.37	87	0.429	0.958	77
ZINC000068198368	0.937	0.621	0.316	128	0.336	0.958	78
ZINC000000156801	0.965	0.563	0.402	75	0.396	0.958	79
ZINC000068247372	0.994	0.664	0.33	48	0.294	0.956	80
ZINC000003950132	0.877	0.706	0.171	310	0.251	0.952	81
ZINC000014960644	1.251	0.808	0.443	4	0.142	0.951	82
ZINC000006716839	0.985	0.664	0.321	54	0.285	0.949	83
ZINC000095616599	0.902	0.787	0.115	208	0.244	0.947	84
ZINC000095641921	0.842	0.574	0.268	472	0.385	0.947	85
ZINC000001159030	0.856	0.575	0.281	396	0.375	0.946	86
ZINC000068249164	0.857	0.59	0.268	389	0.368	0.945	87
ZINC000000355432	0.883	0.583	0.3	289	0.368	0.942	88
ZINC000000000099	0.972	0.583	0.389	66	0.377	0.938	89
ZINC000001753799	0.864	0.507	0.356	366	0.418	0.928	90
ZINC000000009851	0.863	0.561	0.302	370	0.366	0.927	91
ZINC000001530753	0.842	0.543	0.299	474	0.402	0.925	92
ZINC000035793138	0.923	0.646	0.277	167	0.277	0.923	93
ZINC000013983251	0.842	0.679	0.163	469	0.243	0.922	94
ZINC000066097792	0.877	0.692	0.185	311	0.22	0.921	95
ZINC000028950516	0.935	0.765	0.17	132	0.155	0.92	96
ZINC000000008794	0.973	0.687	0.286	63	0.231	0.918	97
ZINC000013130939	0.863	0.712	0.151	369	0.207	0.918	98
ZINC000000057060	0.858	0.582	0.276	385	0.333	0.916	99
ZINC000003955689	0.925	0.738	0.187	158	0.23	0.914	100
ZINC000013916763	0.885	0.589	0.296	275	0.322	0.911	101
ZINC000003881345	1.017	0.687	0.331	32	0.22	0.907	102
ZINC000003986227	0.969	0.526	0.443	69	0.387	0.904	103
ZINC000090470936	0.889	0.618	0.271	255	0.326	0.903	104
ZINC000096024635	0.93	0.685	0.245	144	0.217	0.902	105
ZINC000043178353	1.071	0.725	0.346	14	0.176	0.901	106
ZINC000043208325	0.837	0.614	0.223	495	0.286	0.9	107
ZINC000000070468	0.873	0.582	0.291	329	0.318	0.9	108
ZINC000003875333	0.896	0.645	0.25	233	0.253	0.899	109
ZINC000003683815	1.247	0.76	0.488	5	0.134	0.894	110
ZINC000037866091	0.858	0.698	0.16	387	0.174	0.891	111
ZINC000003950115	0.876	0.613	0.264	313	0.277	0.89	112
ZINC000008648798	1.013	0.7	0.313	34	0.189	0.89	113

ZINC000004212809	0.844	0.569	0.275	461	0.318	0.887	114
ZINC000010773901	0.99	0.505	0.484	52	0.351	0.886	115
ZINC000000607803	0.962	0.576	0.386	78	0.32	0.885	116
ZINC000003821675	0.873	0.682	0.191	330	0.203	0.885	117
ZINC000000386312	0.865	0.556	0.309	359	0.378	0.884	118
ZINC000003873638	0.839	0.675	0.164	487	0.208	0.884	119
ZINC000003966300	0.869	0.602	0.267	339	0.286	0.883	120
ZINC000000652090	0.86	0.557	0.303	379	0.326	0.883	121
ZINC000001533102	0.913	0.48	0.433	186	0.427	0.882	122
ZINC000000538650	0.842	0.658	0.185	467	0.232	0.88	123
ZINC000096032874	0.879	0.591	0.288	300	0.295	0.878	124
ZINC000096283078	0.905	0.594	0.311	200	0.283	0.876	125
ZINC000003812944	1	0.561	0.439	46	0.315	0.876	126
ZINC000003243391	0.987	0.598	0.389	53	0.296	0.873	127
ZINC000003915645	1.029	0.634	0.394	27	0.218	0.873	128
ZINC000000538564	0.84	0.677	0.164	481	0.196	0.873	129
ZINC000000013936	0.978	0.708	0.27	58	0.148	0.872	130
ZINC000043195312	0.97	0.746	0.224	68	0.122	0.871	131
ZINC000043205344	0.893	0.539	0.354	244	0.332	0.871	132
ZINC000000005608	0.844	0.557	0.287	459	0.313	0.87	133
ZINC000169324353	0.844	0.55	0.294	456	0.355	0.869	134
ZINC000008994439	0.927	0.667	0.26	152	0.203	0.869	135
ZINC000043195311	0.97	0.746	0.224	67	0.121	0.868	136
ZINC000040899447	0.925	0.519	0.406	157	0.347	0.866	137
ZINC000096170461	0.933	0.679	0.253	136	0.19	0.866	138
ZINC000059182076	0.938	0.592	0.346	125	0.269	0.862	139
ZINC000035996916	0.87	0.687	0.183	337	0.19	0.861	140
ZINC000000898145	0.838	0.699	0.139	491	0.161	0.86	141
ZINC000012503187	0.903	0.557	0.346	204	0.302	0.858	142
ZINC000035323148	0.934	0.631	0.303	134	0.243	0.858	143
ZINC000000003803	0.941	0.659	0.281	119	0.198	0.857	144
ZINC000008648797	0.969	0.535	0.434	70	0.32	0.855	145
ZINC000003875334	0.928	0.649	0.279	148	0.201	0.851	146
ZINC000095616578	0.948	0.607	0.341	104	0.245	0.851	147
ZINC000002719689	0.952	0.726	0.226	96	0.13	0.847	148
ZINC000012373493	0.944	0.698	0.246	115	0.151	0.846	149
ZINC000043024000	0.899	0.552	0.347	215	0.292	0.844	150
ZINC000001752601	0.878	0.518	0.36	303	0.321	0.844	151
ZINC000000538415	0.877	0.738	0.139	309	0.103	0.841	152

ZINC000001530940	0.919	0.691	0.228	175	0.149	0.841	153
ZINC000095641920	0.839	0.554	0.286	486	0.289	0.84	154
ZINC000068246140	0.881	0.73	0.151	291	0.109	0.839	155
ZINC000143139995	0.905	0.549	0.356	198	0.29	0.839	156
ZINC000001997139	0.899	0.544	0.356	214	0.328	0.837	157
ZINC000001238258	0.852	0.668	0.184	414	0.169	0.837	158
ZINC000019796080	0.948	0.711	0.237	103	0.125	0.836	159
ZINC000004148260	0.867	0.503	0.364	347	0.331	0.835	160
ZINC000115706242	0.88	0.604	0.277	296	0.229	0.833	161
ZINC000000156792	0.865	0.653	0.212	360	0.179	0.832	162
ZINC000040917821	0.897	0.614	0.283	225	0.217	0.832	163
ZINC000068249161	0.854	0.569	0.286	407	0.266	0.831	164
ZINC000175660737	0.844	0.707	0.137	452	0.123	0.83	165
ZINC000019203855	1.06	0.758	0.302	16	0.069	0.828	166
ZINC000007485953	0.908	0.666	0.242	190	0.178	0.828	167
ZINC000034660430	0.887	0.61	0.278	262	0.216	0.825	168
ZINC000001542392	0.867	0.724	0.143	349	0.129	0.824	169
ZINC000097306095	1.009	0.755	0.254	38	0.065	0.82	170
ZINC000000538337	0.963	0.735	0.228	77	0.083	0.818	171
ZINC000001540228	0.936	0.545	0.391	130	0.309	0.818	172
ZINC000224698038	0.969	0.732	0.237	71	0.077	0.818	173
ZINC000013829356	0.837	0.594	0.243	499	0.231	0.817	174
ZINC000095578996	0.881	0.59	0.291	294	0.226	0.816	175
ZINC000029046873	0.992	0.695	0.297	49	0.121	0.815	176
ZINC000040430143	0.879	0.637	0.243	299	0.178	0.815	177
ZINC000013129998	0.848	0.583	0.265	432	0.237	0.813	178
ZINC000003812903	0.886	0.595	0.29	269	0.216	0.812	179
ZINC000096284476	0.858	0.608	0.25	386	0.207	0.811	180
ZINC000073224167	0.842	0.497	0.344	476	0.315	0.811	181
ZINC000000630739	0.892	0.611	0.281	247	0.197	0.808	182
ZINC000003874496	1.048	0.559	0.489	19	0.162	0.807	183
ZINC000040951471	0.854	0.585	0.269	406	0.221	0.807	184
ZINC000001850376	0.888	0.69	0.198	258	0.115	0.805	185
ZINC000003794575	0.886	0.706	0.18	270	0.168	0.805	186
ZINC000059047060	0.838	0.72	0.118	490	0.085	0.805	187
ZINC000033359230	0.944	0.66	0.284	114	0.136	0.804	188
ZINC000003816310	0.897	0.736	0.162	224	0.068	0.804	189
ZINC000043204023	0.937	0.637	0.3	127	0.166	0.804	190
ZINC000003817467	0.959	0.739	0.219	84	0.064	0.804	191

ZINC000003815901	0.886	0.72	0.166	266	0.08	0.8	192
ZINC000034892269	1.007	0.774	0.233	39	0.019	0.798	193
ZINC000003964325	0.891	0.675	0.216	250	0.122	0.797	194
ZINC000003873817	0.968	0.714	0.255	72	0.083	0.796	195
ZINC000012339777	0.869	0.744	0.125	342	0.055	0.796	196
ZINC000003964126	0.94	0.599	0.341	120	0.197	0.795	197
ZINC000100041912	0.861	0.647	0.214	374	0.143	0.793	198
ZINC000102930548	0.85	0.71	0.14	423	0.094	0.793	199
ZINC000004217017	0.907	0.692	0.215	194	0.085	0.793	200
ZINC000003929366	0.948	0.669	0.278	105	0.123	0.792	201
ZINC000029039424	1.035	0.594	0.441	23	0.209	0.79	202
ZINC000058660702	0.884	0.688	0.196	283	0.1	0.79	203
ZINC000003938686	0.877	0.751	0.127	305	0.036	0.789	204
ZINC000252670601	0.864	0.756	0.108	361	0.024	0.788	205
ZINC000003954923	0.925	0.626	0.299	159	0.16	0.786	206
ZINC000001488366	0.918	0.534	0.383	178	0.252	0.786	207
ZINC000049069486	0.897	0.774	0.123	230	0.011	0.785	208
ZINC000002803051	0.905	0.582	0.323	197	0.202	0.784	209
ZINC000043013490	0.885	0.538	0.347	274	0.248	0.783	210
ZINC000000016154	1.057	0.684	0.373	17	0.098	0.782	211
ZINC000210522261	0.955	0.805	0.15	91	-0.023	0.782	212
ZINC000003936850	0.843	0.538	0.305	464	0.262	0.781	213
ZINC000013527116	0.954	0.724	0.231	92	0.056	0.78	214
ZINC000019594594	0.93	0.633	0.297	143	0.147	0.78	215
ZINC000003649002	0.866	0.587	0.28	353	0.216	0.779	216
ZINC000013282483	0.93	0.694	0.237	139	0.085	0.778	217
ZINC000101102775	0.881	0.759	0.123	293	0.017	0.775	218
ZINC000103252336	0.945	0.795	0.15	113	0.002	0.775	219
ZINC000114879021	0.894	0.582	0.312	237	0.185	0.775	220
ZINC000095642810	0.947	0.744	0.203	106	0.03	0.774	221
ZINC000089630340	0.866	0.673	0.193	356	0.101	0.774	222
ZINC000028652818	0.837	0.73	0.107	494	0.043	0.773	223
ZINC000028879084	0.846	0.552	0.295	442	0.198	0.773	224
ZINC000000007761	0.876	0.689	0.187	312	0.082	0.772	225
ZINC000006717791	0.951	0.627	0.325	98	0.145	0.77	226
ZINC000070466423	0.852	0.544	0.307	415	0.224	0.769	227
ZINC000028638129	0.876	0.722	0.154	318	0.058	0.768	228
ZINC000224698099	0.923	0.681	0.243	166	0.077	0.767	229
ZINC000001851251	1.033	0.712	0.321	26	0.055	0.767	230

ZINC000000020221	0.847	0.537	0.31	441	0.221	0.767	231
ZINC000000608261	0.956	0.616	0.34	89	0.159	0.767	232
ZINC000043207516	0.893	0.67	0.223	241	0.096	0.766	233
ZINC000003956919	0.841	0.614	0.227	478	0.098	0.766	234
ZINC000013831810	0.883	0.579	0.304	287	0.178	0.766	235
ZINC000013831229	0.855	0.592	0.263	402	0.173	0.765	236
ZINC000043206271	0.842	0.637	0.206	466	0.128	0.765	237
ZINC000001482913	0.889	0.638	0.251	253	0.124	0.762	238
ZINC000003816409	0.924	0.644	0.28	163	0.119	0.762	239
ZINC000103266485	0.871	0.681	0.19	334	0.083	0.762	240
ZINC000205771844	0.862	0.738	0.124	372	0.023	0.761	241
ZINC000019144231	0.837	0.746	0.091	496	0.013	0.759	242
ZINC000053084692	0.933	0.767	0.165	135	-0.006	0.758	243
ZINC000003810860	0.897	0.588	0.309	227	0.17	0.757	244
ZINC000052716421	1.007	0.772	0.235	40	-0.017	0.755	245
ZINC000005141737	0.897	0.664	0.233	226	0.076	0.754	246
ZINC000000056474	0.838	0.719	0.119	488	0.046	0.752	247
ZINC000027748688	0.886	0.609	0.277	267	0.14	0.748	248
ZINC000000004041	0.982	0.683	0.299	55	0.065	0.748	249
ZINC000001490484	0.884	0.669	0.216	279	0.074	0.748	250
ZINC000028863086	0.92	0.663	0.256	173	0.107	0.748	251
ZINC000001550499	0.936	0.713	0.223	129	0.034	0.748	252
ZINC000000057522	0.894	0.693	0.2	239	0.053	0.746	253
ZINC000001891034	0.927	0.613	0.314	151	0.132	0.746	254
ZINC000003943521	0.851	0.647	0.205	417	0.096	0.743	255
ZINC000091302201	0.891	0.664	0.227	249	0.076	0.74	256
ZINC000000620751	0.929	0.719	0.21	146	0.02	0.739	257
ZINC000003616630	0.939	0.671	0.267	123	0.048	0.739	258
ZINC000038214379	0.924	0.574	0.35	162	0.165	0.739	259
ZINC000034951302	0.877	0.662	0.214	307	0.076	0.739	260
ZINC000095921314	0.901	0.71	0.191	211	0.028	0.739	261
ZINC000000000817	0.887	0.597	0.29	265	0.141	0.738	262
ZINC000095641929	0.893	0.666	0.227	245	0.072	0.737	263
ZINC000001850377	0.881	0.637	0.244	292	0.099	0.736	264
ZINC000098023188	0.899	0.63	0.269	218	0.105	0.735	265
ZINC000068267049	0.938	0.731	0.207	124	0.003	0.734	266
ZINC000008214636	0.952	0.547	0.405	97	0.187	0.734	267
ZINC000306147532	0.928	0.667	0.261	147	0.067	0.734	268
ZINC000009691689	0.928	0.645	0.283	149	0.089	0.734	269

ZINC000049036447	0.908	0.702	0.206	192	0.026	0.733	270
ZINC000001780082	0.838	0.702	0.136	492	0.031	0.733	271
ZINC000003809191	0.856	0.678	0.178	395	0.044	0.732	272
ZINC000000156823	0.887	0.649	0.239	261	0.084	0.732	273
ZINC000043199551	0.899	0.773	0.126	216	-0.002	0.732	274
ZINC000003830507	0.897	0.647	0.25	229	0.088	0.732	275
ZINC000013813297	0.906	0.66	0.247	195	0.064	0.731	276
ZINC000003811810	0.867	0.711	0.156	348	0.012	0.731	277
ZINC000000642589	0.872	0.617	0.255	331	0.113	0.73	278
ZINC000000001115	0.876	0.66	0.216	315	0.069	0.73	279
ZINC000208774715	0.991	0.694	0.298	50	0.059	0.729	280
ZINC000095641932	0.876	0.639	0.236	319	0.087	0.729	281
ZINC000001489478	0.935	0.691	0.243	133	0.037	0.728	282
ZINC000003784077	0.878	0.634	0.244	304	0.094	0.728	283
ZINC000016052239	0.842	0.664	0.178	468	0.064	0.728	284
ZINC000116139756	0.948	0.678	0.271	102	0.05	0.728	285
ZINC000001530694	0.85	0.712	0.138	426	0.011	0.726	286
ZINC000001488270	0.903	0.652	0.251	205	0.076	0.726	287
ZINC000103260612	0.901	0.65	0.251	209	0.075	0.725	288
ZINC000095930152	0.887	0.539	0.348	264	0.191	0.725	289
ZINC000000897322	0.856	0.523	0.332	399	0.201	0.725	290
ZINC000252286763	0.959	0.544	0.416	81	0.163	0.724	291
ZINC000000000471	0.859	0.526	0.333	383	0.198	0.724	292
ZINC000003990436	0.883	0.647	0.236	288	0.09	0.723	293
ZINC000004097841	0.851	0.641	0.21	420	0.08	0.722	294
ZINC000003946509	1.025	0.441	0.584	29	0.28	0.722	295
ZINC000095550949	0.855	0.624	0.23	404	0.097	0.721	296
ZINC000014976393	0.841	0.676	0.165	479	0.045	0.721	297
ZINC000002596977	0.93	0.613	0.316	142	0.109	0.721	298
ZINC000019364229	0.869	0.724	0.145	341	0	0.72	299
ZINC000019632628	0.932	0.714	0.218	137	0.002	0.72	300
ZINC000003986735	0.897	0.638	0.259	231	0.082	0.719	301
ZINC000096286386	0.885	0.716	0.169	276	-0.002	0.718	302
ZINC000040163635	0.937	0.687	0.25	126	0.03	0.718	303
ZINC000003881556	0.846	0.699	0.147	444	0.018	0.717	304
ZINC000013829441	0.936	0.763	0.173	131	-0.046	0.717	305
ZINC000009212278	0.93	0.659	0.271	140	0.057	0.716	306
ZINC000101102777	0.893	0.745	0.148	242	-0.029	0.716	307
ZINC000000014669	0.913	0.637	0.276	185	0.065	0.716	308

ZINC000008214722	0.892	0.675	0.217	246	0.04	0.716	309
ZINC000008781115	0.884	0.572	0.312	277	0.143	0.715	310
ZINC000013675264	0.861	0.627	0.234	376	0.088	0.715	311
ZINC000140341381	1.033	0.593	0.441	24	0.122	0.714	312
ZINC000070647144	0.898	0.613	0.285	223	0.109	0.714	313
ZINC000101581684	1.027	0.734	0.293	28	-0.027	0.711	314
ZINC000036382102	0.877	0.695	0.182	308	0.011	0.711	315
ZINC000072266314	0.98	0.658	0.323	56	0.052	0.71	316
ZINC000003798603	0.844	0.655	0.189	454	0.054	0.709	317
ZINC000003982174	0.843	0.654	0.189	463	0.054	0.707	318
ZINC000000000391	0.912	0.626	0.287	187	0.081	0.707	319
ZINC000096174616	0.979	0.713	0.266	57	-0.007	0.705	320
ZINC000086003193	0.898	0.679	0.22	220	0.025	0.704	321
ZINC000003872044	0.888	0.665	0.224	257	0.04	0.702	322
ZINC000000608382	0.896	0.701	0.195	234	0	0.7	323
ZINC000028332661	0.949	0.659	0.291	100	0.04	0.7	324
ZINC000095930190	0.849	0.694	0.155	428	0.022	0.698	325
ZINC000002559943	0.947	0.669	0.278	108	0.027	0.697	326
ZINC000003876186	0.891	0.691	0.2	251	0.005	0.696	327
ZINC000002541693	0.853	0.658	0.195	410	0.046	0.695	328
ZINC000003812908	0.948	0.612	0.336	101	0.082	0.695	329
ZINC000142095785	0.837	0.542	0.296	493	0.153	0.695	330
ZINC000100015335	0.86	0.673	0.187	378	0.028	0.695	331
ZINC000001489816	0.965	0.74	0.225	74	-0.046	0.695	332
ZINC000003797541	0.838	0.673	0.165	489	0.02	0.694	333
ZINC000000966859	0.962	0.73	0.232	79	-0.036	0.694	334
ZINC000000014551	0.913	0.649	0.264	183	0.045	0.694	335
ZINC000147228368	0.959	0.733	0.226	83	-0.039	0.694	336
ZINC000000057483	0.886	0.688	0.198	268	-0.001	0.694	337
ZINC000000601255	0.85	0.649	0.202	422	0.047	0.694	338
ZINC000000523926	0.867	0.631	0.236	350	0.063	0.693	339
ZINC000095641931	0.906	0.66	0.246	196	0.027	0.692	340
ZINC000095644293	0.837	0.684	0.153	497	0	0.692	341
ZINC000003620786	0.887	0.727	0.16	263	-0.036	0.692	342
ZINC000000008960	0.842	0.593	0.249	471	0.09	0.692	343
ZINC000029043262	0.857	0.592	0.264	394	0.07	0.69	344
ZINC000096032872	0.864	0.595	0.27	362	0.034	0.689	345
ZINC000009212098	0.875	0.506	0.368	321	0.199	0.689	346
ZINC000043207266	0.946	0.537	0.41	112	0.152	0.688	347

ZINC000040424141	0.84	0.647	0.193	485	0.039	0.686	348
ZINC000036520254	0.877	0.745	0.132	306	-0.066	0.685	349
ZINC000004216893	0.851	0.583	0.268	421	0.101	0.684	350
ZINC000040880920	0.843	0.695	0.147	465	-0.011	0.684	351
ZINC000003986651	0.854	0.537	0.317	409	0.129	0.683	352
ZINC000038253214	0.865	0.575	0.291	358	0.108	0.683	353
ZINC000003813328	0.961	0.745	0.216	80	-0.07	0.68	354
ZINC000052541473	0.896	0.704	0.192	232	-0.024	0.68	355
ZINC000036380977	0.914	0.684	0.23	181	-0.006	0.68	356
ZINC000096170449	0.873	0.625	0.248	328	0.054	0.68	357
ZINC000003831238	0.883	0.655	0.229	286	0.025	0.679	358
ZINC000098214604	0.874	0.663	0.211	324	0.014	0.678	359
ZINC000027748678	0.864	0.612	0.253	363	0.067	0.677	360
ZINC000034451922	0.855	0.636	0.218	405	0.029	0.674	361
ZINC000068207701	0.867	0.681	0.187	346	-0.006	0.674	362
ZINC000003779067	0.928	0.708	0.22	150	-0.014	0.673	363
ZINC000000523925	0.863	0.628	0.235	368	0.045	0.673	364
ZINC000071257465	0.847	0.672	0.176	438	0	0.672	365
ZINC000012187083	0.86	0.546	0.313	380	0.124	0.671	366
ZINC000100035725	0.848	0.672	0.176	431	-0.008	0.671	367
ZINC000043207267	0.947	0.537	0.41	109	0.133	0.67	368
ZINC000000001023	0.924	0.637	0.286	165	0.032	0.669	369
ZINC000040414458	0.922	0.568	0.353	172	0.101	0.669	370
ZINC000068250459	0.951	0.629	0.322	99	0.039	0.668	371
ZINC000000599734	0.901	0.757	0.144	210	-0.09	0.667	372
ZINC000013827564	0.836	0.627	0.209	500	0.039	0.666	373
ZINC000000603833	0.856	0.606	0.25	397	0.023	0.666	374
ZINC000001846128	0.859	0.595	0.264	381	0.061	0.664	375
ZINC000021992967	0.868	0.664	0.205	343	0	0.664	376
ZINC000071773472	0.922	0.722	0.199	170	-0.059	0.664	377
ZINC000003875368	0.88	0.557	0.324	295	0.119	0.663	378
ZINC000068245020	0.846	0.61	0.235	446	0.053	0.663	379
ZINC000100907004	0.849	0.613	0.236	429	0.05	0.663	380
ZINC000040414461	0.904	0.562	0.342	203	0.102	0.663	381
ZINC000000602902	0.848	0.547	0.301	433	0.115	0.662	382
ZINC000000057422	0.852	0.567	0.285	413	0.071	0.662	383
ZINC000000608233	0.912	0.674	0.237	189	-0.008	0.661	384
ZINC000082158487	0.869	0.697	0.171	340	-0.037	0.66	385
ZINC000019606670	0.956	0.53	0.426	86	0.106	0.659	386

ZINC000011617039	0.84	0.691	0.149	482	-0.032	0.659	387
ZINC000095583412	0.896	0.696	0.2	235	-0.037	0.659	388
ZINC000003871842	0.922	0.587	0.335	171	0.071	0.658	389
ZINC000038995988	0.924	0.637	0.286	164	0.02	0.657	390
ZINC000070466461	0.875	0.535	0.34	322	0.116	0.657	391
ZINC000001481956	0.927	0.615	0.312	153	0.042	0.657	392
ZINC000013108863	0.848	0.672	0.176	434	-0.015	0.656	393
ZINC000096927633	0.884	0.684	0.2	282	-0.048	0.656	394
ZINC000028049103	0.866	0.668	0.199	355	-0.012	0.655	395
ZINC000009330879	0.857	0.56	0.297	393	0.099	0.655	396
ZINC000006017816	0.9	0.683	0.217	213	-0.034	0.655	397
ZINC000001481805	0.967	0.742	0.225	73	-0.087	0.655	398
ZINC000114456300	0.85	0.615	0.235	425	0.04	0.654	399
ZINC000091291806	0.843	0.654	0.189	462	0	0.653	400
ZINC000004544878	0.88	0.521	0.359	297	0.132	0.653	401
ZINC000019594547	0.845	0.579	0.266	450	0.07	0.652	402
ZINC000096177816	0.857	0.589	0.268	391	0.037	0.652	403
ZINC000003918428	0.85	0.62	0.23	424	0.031	0.651	404
ZINC000100015134	0.868	0.597	0.271	345	0.052	0.649	405
ZINC000019862634	0.894	0.568	0.325	238	0.082	0.649	406
ZINC000000537822	0.885	0.626	0.26	271	0.023	0.649	407
ZINC000001481966	0.854	0.605	0.249	408	0.05	0.649	408
ZINC000084758480	0.872	0.689	0.183	332	-0.032	0.648	409
ZINC000001709414	0.977	0.63	0.347	60	0.018	0.648	410
ZINC000000001894	0.846	0.612	0.234	443	0.036	0.648	411
ZINC000001530811	0.927	0.596	0.33	154	0.05	0.648	412
ZINC000000538202	0.888	0.558	0.33	256	0.088	0.646	413
ZINC000000538483	0.939	0.675	0.264	121	-0.044	0.646	414
ZINC000001601437	0.841	0.59	0.251	477	0.045	0.645	415
ZINC000001530977	0.882	0.675	0.207	290	-0.033	0.642	416
ZINC000001999404	0.947	0.51	0.438	107	0.132	0.642	417
ZINC000003871701	0.864	0.547	0.317	364	0.091	0.642	418
ZINC000000597112	0.842	0.608	0.234	470	0.032	0.64	419
ZINC000095837013	0.861	0.664	0.197	373	-0.026	0.639	420
ZINC000004521526	0.845	0.563	0.283	448	0.079	0.639	421
ZINC000052634547	0.855	0.628	0.227	401	0.011	0.639	422
ZINC000004475069	0.856	0.644	0.211	398	-0.01	0.637	423
ZINC000001489208	0.844	0.608	0.236	457	0.03	0.635	424
ZINC000095828732	0.884	0.485	0.399	285	0.149	0.634	425

ZINC000001530736	0.849	0.584	0.264	430	0.036	0.634	426
ZINC000000001427	1.009	0.632	0.377	37	0.001	0.633	427
ZINC000005442490	0.855	0.582	0.273	403	0.05	0.633	428
ZINC000018710082	0.857	0.543	0.314	392	0.084	0.632	429
ZINC000016052569	0.861	0.683	0.178	377	-0.084	0.631	430
ZINC000003820043	0.889	0.636	0.253	254	0.014	0.63	431
ZINC000014974132	0.875	0.71	0.166	320	-0.081	0.629	432
ZINC000001489490	0.874	0.651	0.223	326	-0.022	0.629	433
ZINC000068200690	1.003	0.704	0.299	43	-0.075	0.629	434
ZINC000009330880	0.876	0.592	0.284	314	0.036	0.629	435
ZINC000058581064	0.898	0.638	0.26	221	-0.011	0.627	436
ZINC000097758762	0.884	0.672	0.212	284	-0.046	0.625	437
ZINC000016052630	0.861	0.629	0.232	375	-0.004	0.625	438
ZINC000019594545	0.841	0.574	0.267	480	0.064	0.623	439
ZINC000019632614	0.869	0.589	0.28	338	0.027	0.622	440
ZINC000004521845	0.955	0.65	0.305	90	-0.014	0.622	441
ZINC000114483165	0.89	0.61	0.28	252	0.011	0.621	442
ZINC000043100956	0.953	0.759	0.194	95	-0.139	0.62	443
ZINC000004214700	0.925	0.561	0.364	161	0.058	0.619	444
ZINC000210248763	0.868	0.664	0.204	344	-0.046	0.618	445
ZINC000035836133	0.944	0.654	0.29	116	-0.036	0.618	446
ZINC000003876078	0.908	0.598	0.311	191	0.02	0.617	447
ZINC000084466381	0.939	0.659	0.28	122	-0.037	0.616	448
ZINC000025783468	0.844	0.588	0.256	455	0.012	0.616	449
ZINC000000005423	0.879	0.661	0.218	301	-0.046	0.615	450
ZINC000084703540	0.85	0.65	0.2	427	-0.014	0.615	451
ZINC000000608172	0.865	0.598	0.267	357	0.016	0.615	452
ZINC000043201623	0.973	0.697	0.276	65	-0.087	0.613	453
ZINC000003822702	0.894	0.638	0.256	240	-0.026	0.612	454
ZINC000001846129	0.848	0.57	0.278	436	0.041	0.61	455
ZINC000041739029	0.902	0.62	0.282	206	-0.064	0.61	456
ZINC000095921739	0.851	0.57	0.281	418	0.036	0.607	457
ZINC000068199903	0.884	0.681	0.204	280	-0.081	0.6	458
ZINC000001489997	0.893	0.713	0.18	243	-0.112	0.6	459
ZINC000013831791	0.874	0.694	0.18	323	-0.094	0.6	460
ZINC000072317687	0.844	0.686	0.158	458	-0.128	0.595	461
ZINC000053045055	0.876	0.591	0.285	317	0.003	0.594	462
ZINC000100036051	0.902	0.461	0.441	207	0.17	0.594	463
ZINC000000642574	0.93	0.563	0.367	141	0.03	0.593	464

ZINC000004688072	0.885	0.601	0.284	273	-0.019	0.586	465
ZINC000062260292	0.859	0.701	0.158	382	-0.115	0.586	466
ZINC000115705166	0.9	0.58	0.321	212	0.006	0.585	467
ZINC000000968328	0.926	0.629	0.297	155	-0.067	0.583	468
ZINC000000276737	0.904	0.465	0.439	201	0.188	0.583	469
ZINC000063539381	0.885	0.657	0.228	272	-0.08	0.577	470
ZINC000003950145	0.846	0.578	0.268	447	-0.006	0.572	471
ZINC000000968327	0.855	0.651	0.205	400	-0.082	0.569	472
ZINC000000007455	0.87	0.583	0.287	336	-0.019	0.564	473
ZINC000006631357	0.922	0.625	0.297	169	-0.063	0.562	474
ZINC000094566093	0.941	0.722	0.219	118	-0.164	0.559	475
ZINC000013985228	0.874	0.696	0.177	325	-0.144	0.552	476
ZINC000043207851	0.851	0.623	0.228	416	-0.076	0.55	477
ZINC000004716567	0.876	0.652	0.224	316	-0.1	0.548	478
ZINC000043206985	0.842	0.692	0.149	473	-0.164	0.545	479
ZINC000118401631	0.899	0.707	0.192	219	-0.163	0.544	480
ZINC000210903069	0.845	0.651	0.194	449	-0.115	0.544	481
ZINC000100015491	0.846	0.584	0.262	445	-0.046	0.538	482
ZINC000001854181	0.858	0.551	0.307	384	-0.016	0.535	483
ZINC000000968330	0.84	0.564	0.277	483	-0.033	0.53	484
ZINC000100067972	0.863	0.422	0.442	367	0.121	0.527	485
ZINC000072317307	0.847	0.522	0.325	439	0.005	0.526	486
ZINC000022063587	0.848	0.572	0.276	435	-0.012	0.515	487
ZINC000001892860	0.866	0.597	0.27	352	-0.063	0.515	488
ZINC000098208332	0.866	0.588	0.278	354	-0.082	0.506	489
ZINC000004015778	0.954	0.604	0.35	93	-0.115	0.489	490
ZINC000000968326	0.844	0.559	0.286	453	-0.07	0.488	491
ZINC000116473771	0.958	0.585	0.373	85	-0.098	0.487	492
ZINC000001542894	0.871	0.439	0.432	333	0.023	0.485	493
ZINC000003932831	0.858	0.616	0.242	388	-0.141	0.475	494
ZINC000006718442	0.853	0.656	0.196	412	-0.187	0.47	495
ZINC000000003381	0.848	0.443	0.405	437	0.022	0.465	496
ZINC000051133897	0.837	0.571	0.266	498	-0.106	0.465	497
ZINC000053294258	0.845	0.546	0.298	451	-0.082	0.464	498
ZINC000040977335	0.844	0.594	0.25	460	-0.125	0.44	499
ZINC000013111071	0.894	0.497	0.397	236	-0.117	0.38	500

Supplementary Table 3

Primary list of drugs tested for their ability to inhibit the store-operated calcium entry pathway

	**Similarity indices of the tested drugs with the cognate query compounds**							
	query: BTP2		query: Pyr6		query: Synta66		query: AnCoA4	
Hit	ROCS rank (TC score)	EON rank (ET combo score)	ROCS rank (TC score)	EON rank (ET combo score)	ROCS rank (TC score)	EON rank (ET combo score)	ROCS rank (TC score)	EON rank (ET combo score)
Leflunomide	1 (1.301)	1 (1.239)	1 (1.265)	1(1.1230)	1 (1.244)	2 (1.20)	368 (0.847)	268 (0.637)
Teriflunomide	48 (0.897)	5(1.036)	14 (0.973)	10 (1.016)	9 (1.003)	7 (1.131)		266 (0.541)
Tolvaptan	10 (0.973)	20 (0.925)	3 (1.063)	6 (1.0600)	2 (1.204)	9 (1.129)	431 (0.83)	68 (0.684)
Conivaptan	155 (0.834)	-	65(0.885)	99 (0.7690)	25 (0.963)	16 (1.057)	575 (0.875)	125(0.564)
Omeprazole	32 (0.913)	42 (0.853)	70(0.882)	4 (1.0860)	33 (0.945)	22 (1.002)	2 (1.087)	1 (1.3)
Lansoprazole	50 (0.895)	72 (0.805)	49(0.901)	291(0.667)	358 (0.811)	-	21 (1.01)	7 (1.034)
Rufinamide	318 (0.79)	311 (0.689)	317(0.789)	253 (0.679)	78 (0.901)	19 (1.03)	-	-
Prazosin	40 (0.901)	132 (0.757)	90(0.866)	131(0.7410)	41 (0.937)	13 (1.073)	19 (1.013)	14 (1.001)
Terazosin	27 (0.915)	7 (1.015)	39(0.918)	7 (1.0420)	10 (1.002)	3 (1.189)	9 (1.041)	13 (1.011)
Flutamide	14 (0.96)	54 (0.84)	10(1.00)	42 (0.876)	92 (0.889)	43 (0.919)	-	-
Roflumilast	2 (1.181)	331 (0.683)	2(1.167)	136 (0.738)	466 (0.793)	194 (0.741)	49 (0.973)	54 (0.853)
